# Rivers across the Siberian Arctic unearth the patterns of carbon release from thawing permafrost

**DOI:** 10.1073/pnas.1811797116

**Published:** 2019-05-06

**Authors:** Birgit Wild, August Andersson, Lisa Bröder, Jorien Vonk, Gustaf Hugelius, James W. McClelland, Wenjun Song, Peter A. Raymond, Örjan Gustafsson

**Affiliations:** ^a^Department of Environmental Science and Analytical Chemistry, Stockholm University, 106 91 Stockholm, Sweden;; ^b^Bolin Centre for Climate Research, Stockholm University, 106 91 Stockholm, Sweden;; ^c^Department of Earth Sciences, Vrije Universiteit Amsterdam, 1081 HV Amsterdam, The Netherlands;; ^d^Department of Physical Geography, Stockholm University, 106 91 Stockholm, Sweden;; ^e^Marine Science Institute, University of Texas at Austin, Port Aransas, TX 78373;; ^f^Yale School of Forestry and Environmental Studies, New Haven, CT 06511

**Keywords:** carbon cycle, climate change, radiocarbon, peat, leaching

## Abstract

Climate warming is expected to mobilize northern permafrost and peat organic carbon (PP-C), yet magnitudes and system specifics of even current releases are poorly constrained. While part of the PP-C will degrade at point of thaw to CO_2_ and CH_4_ to directly amplify global warming, another part will enter the fluvial network, potentially providing a window to observe large-scale PP-C remobilization patterns. Here, we employ a decade-long, high-temporal resolution record of ^14^C in dissolved and particulate organic carbon (DOC and POC, respectively) to deconvolute PP-C release in the large drainage basins of rivers across Siberia: Ob, Yenisey, Lena, and Kolyma. The ^14^C-constrained estimate of export specifically from PP-C corresponds to only 17 ± 8% of total fluvial organic carbon and serves as a benchmark for monitoring changes to fluvial PP-C remobilization in a warming Arctic. Whereas DOC was dominated by recent organic carbon and poorly traced PP-C (12 ± 8%), POC carried a much stronger signature of PP-C (63 ± 10%) and represents the best window to detect spatial and temporal dynamics of PP-C release. Distinct seasonal patterns suggest that while DOC primarily stems from gradual leaching of surface soils, POC reflects abrupt collapse of deeper deposits. Higher dissolved PP-C export by Ob and Yenisey aligns with discontinuous permafrost that facilitates leaching, whereas higher particulate PP-C export by Lena and Kolyma likely echoes the thermokarst-induced collapse of Pleistocene deposits. Quantitative ^14^C-based fingerprinting of fluvial organic carbon thus provides an opportunity to elucidate large-scale dynamics of PP-C remobilization in response to Arctic warming.

## Significance

High-latitude permafrost and peat deposits contain a large reservoir of dormant carbon that, upon warming, may partly degrade to CO_2_ and CH_4_ at site and may partly enter rivers. Given the scale and heterogeneity of the Siberian Arctic, continent-wide patterns of thaw and remobilization have been challenging to constrain. This study combines a decade-long observational record of ^14^C in organic carbon of four large Siberian rivers with an extensive ^14^C source fingerprint database into a statistical model to provide a quantitative partitioning of the fraction of fluvially mobilized organic carbon that specifically stems from permafrost and peat deposits, and separately for dissolved and particulate vectors, across the Siberian Arctic, revealing distinct spatial and seasonal system patterns in carbon remobilization.

The destabilization of permafrost and peat deposits in a warming Arctic involves a range of mechanisms that act on different temporal and spatial scales. Rising temperatures promote a gradual deepening of the seasonally thawed active layer at the surface of permafrost soils and a decrease in areal permafrost extent at the southern margin of the permafrost zone (1). Rising temperatures and increasing precipitation can further induce abrupt landscape collapse and degradation of deeper organic carbon deposits. Ice-rich permafrost deposits are particularly vulnerable to collapse (thermokarst) (2), including Holocene but also Pleistocene deposits that are still widespread, especially across northeastern Siberia (Ice Complex deposits, or Yedoma) (3). Changing climatic conditions might further destabilize deep peat deposits that have accumulated during the Holocene across the circum-Arctic (4–6). Peat is particularly abundant in the World’s largest wetland—the West Siberian Lowland—which is largely underlain by vulnerable discontinuous permafrost and projected to experience a further decrease in permafrost extent (7).

Permafrost and peat degradation affects vast and remote areas where there is very limited access to field data. To complement existing, rare, and largely point-specific studies across the heterogeneous landscape of the Siberian Arctic and tackle the upscaling challenge, this study employs rivers as natural integrators of carbon mobilization because they transport organic carbon released by abrupt collapse and erosion of old Holocene and Pleistocene deposits, as well as organic carbon leached from deepening active layers, next to organic carbon recently fixed by plants. The different ^14^C ages of these organic carbon sources are used in a fingerprinting approach to distinguish flux components from different permafrost and peat organic carbon (PP-C) pools versus recent primary production.

**Fig. 1.**
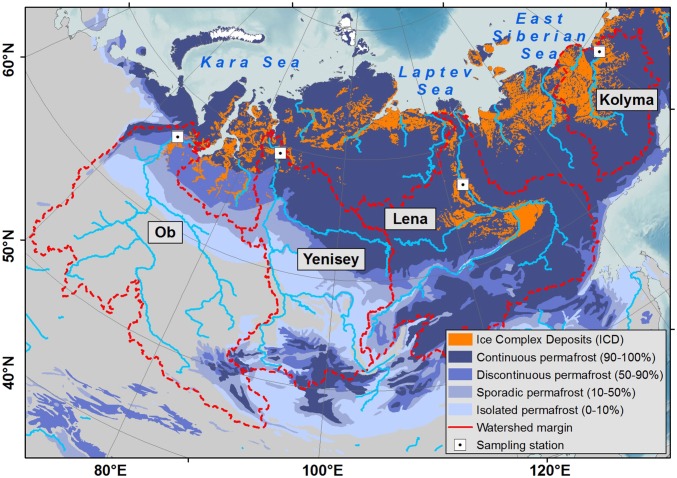
Northeastern Eurasia with watershed margins of the Ob, Yenisey, Lena, and Kolyma rivers, underlain with the spatial extents of continuous, discontinuous, sporadic, and isolated permafrost (60), as well as of Pleistocene Ice Complex deposits (61).

We take advantage of the unique decade-long, high temporal resolution records of organic carbon fluxes and ^14^C contents in the Ob, Yenisey, Lena, and Kolyma [2003–2013; *n* = 110 for particulate organic carbon (POC), *n* = 137 for dissolved organic carbon (DOC) covering all seasons] generated by the river monitoring programs Pan Arctic River Transport of Nutrients, Organic Matter, and Suspended Sediments (PARTNERS) and Arctic Great Rivers Observatory (ARCTIC-GRO) (8). The basins of the four rivers span 110° in longitude and cover a combined area of 8.2 million km^2^, with 5.8 million km^2^ in the northern permafrost region. This area corresponds to 26% of the northern circumpolar permafrost area and 40% of the northern Eurasian permafrost area (Fig. 1). Combining the ^14^C datasets of both POC and DOC with an extensive database on ^14^C fingerprints of the potential organic carbon sources using statistical source apportionment, this study provides a quantitative estimate of fluvial organic carbon export specifically source-apportioned to permafrost and peat deposits and assesses the potential of ^14^C signatures of POC and DOC to monitor changes in PP-C release in a warming climate.

## Results and Discussion

### Preaged Organic Carbon in Siberian Rivers.

**Fig. 2.**
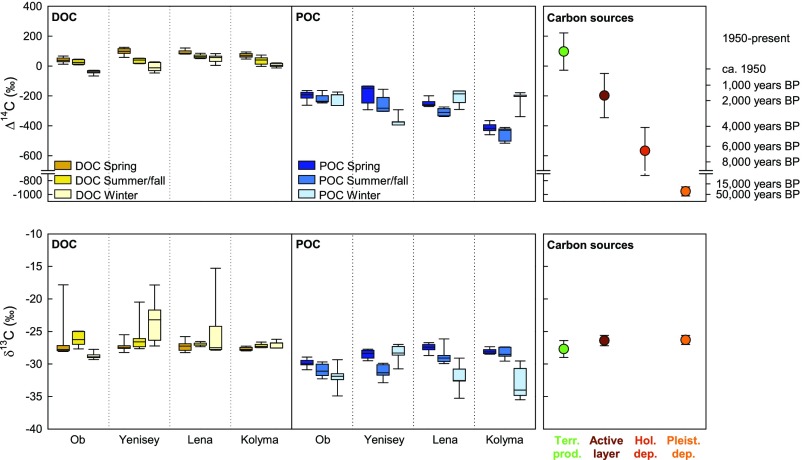
Carbon isotopic composition of DOC (*Left*) and POC (*Center*) in four large Siberian rivers, expressed as Δ^14^C and δ^13^C values. Boxplots show medians with 25th and 75th percentiles as box limits and 10th and 90th percentiles as whiskers, with isotopic data weighted by the flux rate of DOC and POC at the corresponding time point. Endmember Δ^14^C and δ^13^C values (mean ± SD) of potential organic carbon sources (*Right*) indicate recent terrestrial primary production (Terr. prod.), active layer, Holocene deposits (Hol. dep.), and Pleistocene deposits (Pleist. dep.); note that δ^13^C values for Holocene deposits are not shown here; see *SI Appendix*, *Supplementary Information Text* for details. Conventional ^14^C dates derived from Δ^14^C values are indicated at the right margin. BP, before present. Both Δ^14^C and δ^13^C values were significantly lower for POC than for DOC (flux-weighted *t* test, *P* < 0.001; see *SI Appendix*, Table S7 for statistical analyses of differences between rivers and seasons).

All four rivers show significantly lower Δ^14^C values for POC than for DOC (flux-weighted *t* test, *P* < 0.001; Fig. 2), signaling a greater proportion of old carbon in POC, presumably from permafrost and peat deposits (9–11). This observation is in line with previous studies that suggest erosion of deep and consequently old deposits along riverbanks as a large source of POC, but not DOC, to Arctic rivers (12). Across rivers and seasons, POC-Δ^14^C values averaged −261 ± 82‰ (flux-weighted mean ± SD, *n* = 110), corresponding to mean conventional ^14^C ages of 2,400 (SD +900/−800) y. The POC-Δ^14^C values of the eastern-most river, Kolyma, were significantly lower than those of the Lena and of the western rivers, Ob and Yenisey (Fig. 2 and *SI Appendix*, Table S7). This pattern is consistent with a systematic increase in ^14^C ages from west to east along the Eurasian–Arctic continental border that has been previously described for river POC and river delta surface sediments (10, 13, 14) and suggests a greater influence of compartments with high reservoir ages such as the Ice Complex deposits toward the east.

In contrast, more contemporary Δ^14^C values of DOC indicate a relatively higher contribution of recently fixed organic carbon. Across all rivers and seasons, DOC-Δ^14^C values averaged +72 ± 39‰ (flux-weighted mean ± SD, *n* = 137), pointing at a dominance of carbon taken up by plants between the 1950s and the present, when atmospheric CO_2_ was enriched in ^14^C due to nuclear weapons tests (15). In contrast to POC, DOC-Δ^14^C values were lowest in the Ob, followed by the Kolyma and Yenisey as well as the Lena (Fig. 2 and *SI Appendix*, Table S7). The DOC-Δ^14^C values further decreased significantly from spring (ice breakup; May and June) to summer/fall (ice-free period; July through October) and winter (ice-covered period; November through April) in all four rivers.

### Quantifying the Permafrost and Peat Component of Fluvial Carbon Export.

While the ^14^C signatures of POC and DOC in Arctic rivers can give some indication of carbon release from high-latitude permafrost and peat deposits, pinpointing and monitoring the spatial and temporal dynamics of PP-C release is challenging because both POC and DOC represent mixtures of carbon from different sources. Here, isotope-based source apportionment with Markov chain Monte Carlo simulations provides the tool to quantify the relative contribution of PP-C to the total fluvial organic carbon load and thereby isolate the spatial and temporal patterns of PP-C release. This approach allows us to calculate the contribution of different organic carbon sources to river DOC and POC using their Δ^14^C values while accounting for variability in both river observations and source endmember values (16). The Δ^14^C values of recent organic carbon and three potential sources of preaged PP-C were estimated based on an extensive literature review that is described in detail in *SI Appendix*, *Supplementary Information Text*. The Δ^14^C endmember of recent terrestrial primary production was constrained based on observations from litter and organic layers to +97 ± 125‰ (*n* = 58; Fig. 2), reflecting the contemporary to only decades-aged nature of this carbon pool. The three potential preaged PP-C sources were (*i*) the active layer with average Δ^14^C values of −198 ± 148‰ (1,700 y, *n* = 60); (*ii*) Holocene permafrost, peat, and thermokarst deposits with Δ^14^C values of −568 ± 157‰ (6,700 y, *n* = 138); and (*iii*) Pleistocene permafrost deposits such as Ice Complex deposits with Δ^14^C values of −955 ± 66‰ (24,800 y, *n* = 329). For the latter two carbon sources, we specifically considered only exposures along riverbanks and coasts to most realistically represent the Δ^14^C range of material that may enter rivers by erosion. Although the high turbidity in the rivers constrains photosynthesis to a thin layer of surface waters (17), aquatic production by phytoplankton and bacteria can potentially contribute to the total POC load (18). Nevertheless, the mineralization of terrestrial carbon during river transport typically leads to oversaturation in CO_2_ compared with the atmosphere and, thus, to limited influx of atmospheric CO_2_ (19–23). Aquatic production is consequently largely fueled by recycling of terrestrially derived carbon and therefore not considered an independent carbon source here (see also discussion in *Fate of PP-C During River Transport*).

Source apportionment between the recently formed carbon reservoirs versus the preaged PP-C was performed in three scenarios assuming different contributions of active layer, Holocene deposits, and Pleistocene deposits to the PP-C flux (see *SI Appendix*, *Supplementary Information Text* for details). The Best Estimate scenario represents the most realistic and conservative estimate because it assumes a contribution of all PP-C compartments to the PP-C flux and considers the uncertainties of not only individual carbon source Δ^14^C values but also their proportional contribution to the PP-C flux. Pleistocene deposits were considered only for the Lena and Kolyma catchments where these are abundant (Fig. 1). The sensitivity of the model’s results to the assumptions of the Best Estimate scenario was additionally tested in the Maximum and Minimum scenarios assuming a contribution of only the youngest (active layer) or oldest (Ob and Yenisey Holocene deposits, Lena and Kolyma Pleistocene deposits) PP-C compartment, respectively. These scenarios provide an even more conservative uncertainty envelope of the estimated PP-C flux, but are realistic only on very small spatial scales and not on the large scales of the great Siberian rivers that integrate carbon release from various sources within their catchments.

**Fig. 3.**
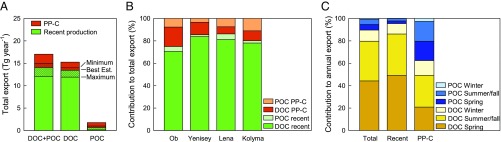
Comparison of recent organic carbon and PP-C fluxes. (*A*) Contribution of PP-C to total organic carbon export in four large Siberian rivers. Fractions of PP-C compared with recent primary production are based on the Best Estimate scenario; the shaded areas indicate the intervals between the Minimum and Maximum scenarios. (*B*) Contribution of carbon from recent primary production and PP-C in dissolved and particulate form to the total organic carbon export in individual rivers (Best Estimate scenario; see *SI Appendix*, Table S9 for other scenarios). (*C*) Contribution of DOC and POC in spring, summer/fall, and winter to the annual export of total organic carbon, recent organic carbon, and PP-C (Best Estimate scenario; see *SI Appendix*, Fig. S2 for other scenarios). Total organic carbon export was quantified based on discharge, POC, and DOC concentration measurements using the LOADEST program (10, 24).

The fluvial PP-C export vector was calculated by combining the ^14^C-constrained contribution of PP-C to total fluvial organic carbon (*SI Appendix*, Table S8) with previous estimates of the total fluvial organic carbon flux load (10, 24) (*SI Appendix*, Table S9). In the Best Estimate scenario, we thus arrive at a (DOC + POC) combined PP-C export of 3.0 ± 0.3 Tg PP-C per year by Ob, Yenisey, Lena, and Kolyma (Minimum: 2.0 ± 0.2 Tg y^−1^; Maximum: 5.0 ± 0.5 Tg y^−1^; Table 1). This first estimate of the deconvoluted PP-C export component for the four largest Siberian rivers represents a benchmark for monitoring the fluvial remobilization of preaged organic carbon from permafrost and peat deposits in a warming climate, and contributes toward a three-dimensional, quantitative understanding of Arctic carbon cycling that considers not only vertical but also lateral fluxes. Our estimate demonstrates that the PP-C component corresponds to only 17 ± 8% of the total fluvial organic carbon load of 17.0 ± 1.3 Tg y^−1^ (Minimum: 12 ± 6%; Maximum: 29 ± 11%; Fig. 3*A*). Hence, this study reveals that patterns of fluvial PP-C remobilization are masked by large fluxes of recent organic carbon; quantitatively dissecting the PP-C flux, however, opens an observational window to monitor PP-C release across heterogeneous landscapes and seasons and thereby advance our understanding of PP-C vulnerability.

**Table 1.** Estimates of total organic carbon (DOC + POC) fluxes in Ob, Yenisey, Lena, and Kolyma, as well as carbon fluxes from permafrost and peat deposits

River	Total organic carbon* (Tg y^−1^)	PP-C^†^ (Tg y^−1^)		
		Best Estimate	Minimum	Maximum
Ob	4.7 ± 0.7	1.2 ± 0.2	0.9 ± 0.1	1.8 ± 0.3
Yenisey	4.9 ± 0.4	0.7 ± 0.2	0.5 ± 0.1	1.1 ± 0.2
Lena	6.5 ± 1.0	0.9 ± 0.2	0.5 ± 0.1	1.7 ± 0.3
Kolyma	0.9 ± 0.2	0.2 ± 0.0	0.1 ± 0.0	0.3 ± 0.1
All rivers	17.0 ± 1.3	3.0 ± 0.3	2.0 ± 0.2	5.0 ± 0.5

Values are represented as mean ± SD.

* Estimates from refs. 10 and 20.

^†^ Best Estimate, Minimum, and Maximum represent model scenarios for PP-C fluxes. See *SI Appendix*, Table S9 for detailed data.

Although fluvial organic carbon was strongly dominated by DOC (90 ± 4%), all three model scenarios suggest that POC contributed about one-third to one-half of the total PP-C export (Best Estimate: 38 ± 19% of PP-C export in the form of POC; Fig. 3*B* and *SI Appendix*, Fig. S2). While DOC contained mostly recent organic carbon and only 12 ± 8% PP-C, POC carried a strong PP-C ^14^C signature (63 ± 10% of POC from PP-C; Fig. 3*A*). Hence, the ^14^C signature of POC offers the best opportunity to chronicle the dynamics of PP-C remobilization.

### Unearthing the Seasonal and Spatial Dynamics of PP-C Remobilization.

Isotopically deconvoluting recent organic carbon and PP-C contributions to fluvial carbon fluxes provides the opportunity to study the seasonal and spatial dynamics specifically of PP-C in rivers without interference by the much larger flux of recent organic carbon. The PP-C in dissolved and particulate carrier phases showed distinct seasonal patterns that suggest different dominant mobilization pathways (Fig. 3*C*). The contribution of PP-C to DOC increased from spring to summer/fall and winter (*SI Appendix*, Fig. S3 and Table S8), which points to gradual leaching as a key mechanism of dissolved PP-C release. Gradual leaching increasingly mobilizes deeper (and older) organic carbon after top-down soil thaw from spring to summer and refreezing from fall to winter (25, 26), including carbon thawed at the permafrost table by active-layer deepening. Seasonal differences in dissolved PP-C release might also be related to changes in the relative importance of surface runoff and deep groundwater-dominated flow paths in the river catchments (27). Overall, the remobilization of PP-C in dissolved form was delayed compared with that of recent carbon. In contrast, the contribution of PP-C to total POC remained constantly high throughout the year, and no distinct differences in seasonal dynamics of PP-C versus recent carbon were observed (*SI Appendix*, Table S8). Particulate PP-C might therefore, to a larger extent, derive from abrupt collapse of deep Holocene and/or Pleistocene deposits that can instantaneously release organic carbon of various ages into aquatic systems (see refs. 28 and 29); for example, by thermal or ice jam-induced bank erosion (30). Contrasting DOC and POC mobilization pathways have been previously suggested based on the different ^14^C ages of DOC and POC (11, 31); isolating the PP-C component of DOC and POC and pinpointing its seasonal patterns finally confirms gradual leaching and abrupt collapse as the main sources of dissolved and particulate PP-C release, respectively. Overall, the export of recent organic carbon was dominated by the spring freshet, with 52 ± 13% of the annual export in spring, 39 ± 13% in summer/fall, and 10 ± 3% in winter. These findings are in line with previous observations of high lignin concentrations in DOC in spring that suggest strong leaching of recent plant litter during this period (9). The export of PP-C, in contrast, was shifted toward later seasons, with 38 ± 12% of the annual flux in spring, 46 ± 12% in summer/fall, and 16 ± 9% in winter.

Differences in the export of dissolved and particulate PP-C also between rivers provide an opportunity to assess the vulnerability of high-latitude carbon stocks across geospatial climatic gradients. Export of PP-C in Ob and Yenisey was dominated by DOC (Fig. 3*B*). Leaching of organic carbon from soils to streams may be facilitated here by discontinuous permafrost in the Ob and Yenisey catchments (Fig. 1) (14), as well as by large peatlands that provide limited retention of organic carbon by sorption to soil minerals (4, 32). In contrast, a larger fraction of PP-C export by Lena and Kolyma was in particulate form (Fig. 3*B*), reflecting stronger erosion, likely to a large extent of Pleistocene Ice Complex deposits that are abundant in both catchments and particularly susceptible to erosion due to their high ice content (2, 3). Overall, the contribution of PP-C to the total fluvial carbon load was lowest in the Yenisey and increased both toward the Lena and Kolyma in the east and toward the Ob in the west. The Ob showed the strongest PP-C contribution to total export, had the highest rate of PP-C flux, and accounted for 40% of the PP-C exported by the four large Siberian rivers (Table 1).

### Fate of PP-C During River Transport.

Comparing Δ^14^C and δ^13^C values of fluvial organic carbon and its deduced sources may further provide information about the fate of PP-C during river transport. The δ^13^C values of terrestrial carbon pools in the drainage area of the four rivers fall within a narrow range characteristic of dominant C3 vegetation, with estimates of −27.7 ± 1.3‰ for terrestrial primary production, −26.4 ± 0.8‰ for the active layer, and −26.3 ± 0.7‰ for Pleistocene Ice Complex deposits based on previous studies (see *SI Appendix*, *Supplementary Information Text* for details). The δ^13^C values of river DOC were in the same range (−27.0 ± 2.0‰, flux-weighted mean ± SD of all rivers and seasons, *n* = 134; Fig. 2), whereas the δ^13^C values of river POC were significantly lower, with an annual mean of −28.7 ± 1.6‰ (*n* = 162) and a wintertime mean of −31.1 ± 3.2‰ (*n* = 37) despite a predominantly terrestrial ^14^C signature.

Differences in the ^13^C content of POC compared with its source indicate loss of POC during transport. The ^13^C content of organic carbon is therefore not a conservative source marker during long-range aquatic transport. Fluvial organic carbon may be mineralized to dissolved inorganic carbon, which is partly outgassed to the atmosphere and partly taken up by phytoplankton to fuel photosynthesis. Terrestrial carbon may thereby reenter the POC pool in the form of phytoplankton after an additional ^13^C fractionation step, consequently lowering POC δ^13^C values. In line with the recycling of organic carbon during river transport, Ob and Yenisey phytoplankton shows low δ^13^C values (−30.6 ± 3.3‰, *n* = 24; see *SI Appendix*, *Supplementary Information Text* and ref. 33) similar to those of POC. Lower δ^13^C values of phytoplankton have also been observed in the Laptev Sea (which receives strong carbon input from the Lena River) as compared to the East Siberian Sea (34). Degradation of terrestrially derived organic carbon during river transport is further supported by previous incubation studies that show a rapid loss of DOC in permafrost leachates and rivers (35–43), with highest losses of up to 53% within 9 d for DOC from Ice Complex deposits (36, 37, 44). Comparable incubation studies on the degradability of POC are urgently needed, considering the high contribution of POC to PP-C export as demonstrated in this study. In addition to the “fast pool” within terrestrially derived carbon, other fractions will be degraded more slowly [as indicated by CO_2_ oversaturation of river and coastal ocean waters far away from the original source location compared with the atmosphere (23, 45)], or will be resequestered by sedimentation. Applying ^14^C-based source apportionment to DOC and POC at different locations along the fluvial network in future studies could improve our understanding of terrestrial carbon transfer to rivers, the fraction of DOC and POC degraded or sedimented during different stages of aquatic transport, and thereby the “boundless carbon cycle” that connects multiple cooccurring processes on land, in the water, and in the atmosphere.

### The ^14^C Signature of Fluvial Organic Carbon as Indicator of Future PP-C Release.

The ^14^C signature of carbon in large rivers may serve as an indicator to monitor the release of carbon from high-latitude permafrost and peat deposits in a warming climate. The sensitivity of fluvial Δ^14^C values to changes in PP-C release was tested by simulating a decrease or increase in PP-C flux by factors ranging from 0.5 to 2.0. The Best Estimate approach, at constant recent carbon flux, was used for this sensitivity test. The resulting shift in Δ^14^C values of fluvial organic carbon depended on baseline Δ^14^C values (i.e., Δ^14^C values without change in PP-C flux), with highest sensitivity at baseline Δ^14^C values between −100‰ and −200‰ in the western rivers Ob and Yenisey, and between −100‰ and −300‰ in the eastern rivers Lena and Kolyma (*SI Appendix*, Fig. S4). Doubling the PP-C flux resulted in a maximum decrease in Δ^14^C by 82‰ (western rivers) and 109‰ (eastern rivers). Halving the PP-C flux resulted in an increase by the same values.

The suitability of DOC and POC to monitor changes in PP-C release in a warming Arctic depends on (*i*) current DOC-Δ^14^C and POC-Δ^14^C values, including their variability; (*ii*) the sensitivity of Δ^14^C baseline values to changes in PP-C release; and (*iii*) the expected change in PP-C release in dissolved and particulate form. The sensitivity of the DOC pool to resolve changes in PP-C release is challenged by the strong dilution of PP-C with recent carbon in this pool. For Yenisey and Lena, increases in PP-C release by an additional 159% and 130%, respectively, would be required to result in a statistically significant difference (*P* < 0.05) to baseline DOC-Δ^14^C values (*SI Appendix*, Fig. S4). Sensitivity is higher in Ob and Kolyma, with minimum resolvable increases in PP-C release by 27% and 48%, respectively. Considering that gradual active-layer leaching releases mostly dissolved PP-C as indicated by the spatial and temporal dynamics of the isotopically deconvoluted PP-C flux, our findings suggest overall low sensitivity of the fluvial ^14^C signature to active-layer deepening. However, the sensitivity of DOC-Δ^14^C might be sufficient in Ob and Kolyma, as well as in winter when the relative contribution of PP-C reaches its annual maximum and when, especially, the deep active layer may still be unfrozen (25, 26).

By contrast, the ^14^C signature of POC is likely a sensitive indicator of abrupt collapse of deeper PP-C deposits. Particulate PP-C stemmed, to a large extent, from erosion and strongly dominated the total POC flux (63 ± 10% PP-C), resulting in a higher sensitivity of POC-Δ^14^C values compared to DOC-Δ^14^C values (*SI Appendix*, Fig. S4). An increase in PP-C release by only 24 to 36% would thus induce statistically resolvable changes in POC-Δ^14^C in all four rivers (*SI Appendix*, Fig. S4). In comparison, the total fluvial sediment load by erosion has been projected to increase by 30 to 122% until 2100 in the six largest Eurasian rivers, including Ob, Yenisey, Lena, and Kolyma (46), and by 200 to 600% in a smaller river in the Canadian Arctic (47). Both projections are likely minimum estimates because they considered only the changes in general hydrological properties and temperature, not the expected increases in permafrost thaw and thermokarst formation in the river catchments (2). Changes might be even larger if tipping points in Arctic permafrost thawing are passed, as indicated in a recent study (48). Our analyses suggest the high sensitivity of POC-Δ^14^C values to even comparatively moderate increases in PP-C release by erosion in the river catchments; the Δ^14^C values of POC consequently provide the best observational window to detect changes in PP-C release.

## Conclusions

Rising temperatures are expected not only to accelerate the degradation of PP-C to CO_2_ or CH_4_ at point of thaw, thereby inducing a positive feedback to climate warming (49), but also, simultaneously, to alter carbon release into the fluvial network (32, 50). Part of the fluvial carbon will eventually reach the Arctic Ocean where a significant portion is degraded, thousands of kilometers away from point of thaw (51), giving rise to CO_2_ efflux to the atmosphere and severe ocean acidification (45). However, large spatial heterogeneity in organic carbon thaw and remobilization is expected on local, drainage basin, and subcontinental scales (1, 2, 32), implying that complementary approaches to site-specific observations are needed to meet this upscaling challenge.

This study combines an extensive, decade-long set of river observations that integrates release processes over large drainage basins with ^14^C-based source apportionment to provide quantitative constraints on organic carbon mobilization specifically from permafrost and peat deposits across Siberia. Despite the vast extent of old permafrost and peat deposits in the catchments of Ob, Yenisey, Lena, and Kolyma, fluvial organic carbon and, in particular, DOC were strongly dominated by recent primary production. Hence, DOC in Arctic rivers carries limited information on permafrost carbon release. In contrast, POC was dominated by remobilized PP-C (63 ± 10%). Although POC constituted merely 10% of the total fluvial organic carbon load, it thus accounted for more than a third of the fluvial PP-C export and represents the best observational window to monitor PP-C release in a warming climate. Deconvoluting the relative contributions of recent organic carbon versus PP-C fluxes revealed distinct seasonal patterns, with carbon export from recent vegetation dominated by the spring freshet and with PP-C mobilization shifted toward summer, fall, and winter, highlighting the importance of late-season PP-C release processes. Dissolved PP-C export dominated the western rivers Ob and Yenisey whose drainage basins are characterized by less permafrost coverage and less soil carbon retention. In contrast, higher particulate PP-C export in the eastern rivers Lena and Kolyma echoes the thermokarst-induced, abrupt collapse of Pleistocene Ice Complex deposits. Quantitative ^14^C-based fingerprinting of fluvial organic carbon, especially of the POC, thus provides information on changes to the otherwise invisible subsurface cryosphere carbon over large spatial scales in response to Arctic warming and advances our understanding of the inner workings of large-scale permafrost carbon remobilization—an essential component for meaningful predictions of the Arctic PP-C–climate feedbacks.

## Methods

### DOC and POC Sampling and Analyses.

Samples for DOC and POC analyses were collected at Salekhard (Ob), Dudinka (Yenisey), Zhigansk (Lena), and Cherskiy (Kolyma) between July 2003 and November 2013 as part of the PARTNERS and ARCTIC-GRO programs (8). Details on DOC and POC sampling and analysis methods can be found in *SI Appendix*, *Supplementary Information Text*, as well as in previously published papers (10, 24, 52) and in the metadata provided with the publicly available datasets (https://arcticgreatrivers.org/). Original data are presented in *SI Appendix*, Table S6 and on the project homepage (https://arcticgreatrivers.org/), and have been used for other purposes in previous studies (9, 10, 52).

Data were categorized into a spring period (May and June), characterized by ice breakup and high discharge; a combined summer and fall period (July to October), during which rivers are ice-free; and a winter period (November to April), when rivers are ice covered and discharge is low (10, 24). In the case of DOC-Δ^14^C, the spring season included 59 individual data points, the summer/fall included 49, and the winter included 29. In the case of POC-Δ^14^C, spring was represented by 50 data points, summer/fall by 42, and winter by 20. We thereby also captured the spring freshet, when discharge and carbon export can change rapidly at high temporal resolution (*SI Appendix*, Fig. S1). Total DOC and POC export rates in individual seasons and rivers were derived from previous publications based on the PARTNERS/ARCTIC-GRO data on discharge, POC, and DOC concentrations. These studies applied the US Geological Survey Load Estimator (LOADEST) program that builds regression equations to relate variations in flux with variations in discharge over multiple years while accounting for seasonal variations in those relationships. A detailed description of the approach is provided in the original publications (10, 24). Isotopic data were weighted by the corresponding DOC or POC fluxes at the time of sampling to achieve a proportional representation of periods with high and low DOC and POC flux. Percentiles of flux-weighted data for box plots were calculated using the Hmisc package (53) in R 3.5.1 (54). Differences between DOC and POC were tested for significance using *t* tests of flux-weighted data in the weights package (55) in R, and differences between rivers and seasons of flux-weighted data were tested using two-way ANOVA with Tukey’s honest significant difference post hoc test in the HH package (56) in R.

### Source Apportionment and Markov Chain Monte Carlo Simulations.

The contribution of PP-C to DOC and POC was quantified based on the Δ^14^C signatures of four potential organic carbon source pools that were derived from extensive literature review as described in detail in *SI Appendix*, *Supplementary Information Text*, substantially updating previous versions of endmember databases from the Siberian Arctic (57, 58): (*i*) Recent terrestrial primary production Δ^14^C values were estimated as 97.0 ± 124.8‰, based on published data from organic and litter layers in arctic, subarctic, and boreal systems in northern Russia, northern Scandinavia, northern Canada, and Alaska (*n* = 58); (*ii*) Active-layer Δ^14^C values were constrained as −197.5 ± 148.4‰, based on data from active layers and nonpermafrost soils (excluding organic layers) in northern Siberia (*n* = 60); (*iii*) Holocene permafrost, peat, and thermokarst deposits were of similar age and thus combined; mean Δ^14^C values of Holocene deposits were calculated from exposures of peat and thermokarst in northern Siberia as −567.5 ± 156.7‰ (*n* = 138); and (*iv*) Pleistocene deposit Δ^14^C values were estimated as −954.8 ± 65.8‰, based on data from Pleistocene Ice Complex deposit exposures in northeastern Siberia (*n* = 329). Considering the common oversaturation of Arctic rivers with CO_2_ and the consequently limited influx of atmospheric CO_2_ (19–23), aquatic primary production represents largely recycling of terrestrial carbon and is thus not considered an independent organic carbon source. Nevertheless, the ∆^14^C range of atmospheric CO_2_ during the time of sampling of +48 ± 11‰ (59) falls within the range of recent terrestrial primary production; any potential minor influx of atmospheric CO_2_ would therefore be within the uncertainty of the endmember for recent carbon.

Source apportionment was performed for two endmembers that represent recent carbon and PP-C (see *SI Appendix*, *Supplementary Information Text* for details). The Δ^14^C values of the PP-C endmember were calculated for three scenarios assuming different contributions of organic carbon from active layers, Holocene deposits, and Pleistocene deposits to the PP-C flux. The Best Estimate scenario likely represents the most realistic estimate because it considers a contribution of all compartments to the PP-C flux. Specifically, a least-biased approach was used in which all fractional combinations of individual compartments are set to be equally likely. Compared with the assumption of equal contributions, this approach results in the same average Δ^14^C value of the combined PP-C endmember, but also in a larger uncertainty that includes both the uncertainty of individual endmember constraints and the uncertainty of their proportional contribution to the PP-C endmember. Pleistocene deposits were considered only for the Lena and Kolyma catchments where they are abundant (Fig. 1). Model sensitivity was tested in the Maximum and Minimum scenarios assuming a contribution of only the youngest (active layer) or oldest (Ob and Yenisey Holocene deposits, Lena and Kolyma Pleistocene deposits) PP-C compartment, respectively. To account for the variability of the endmembers, the relative source contribution estimates were calculated within a Bayesian Markov chain Monte Carlo framework (16). The simulations were run in MATLAB (version 2014b), using 1,000,000 iterations, a burn-in phase of 10,000, and a data thinning of 10.

### Data Availability.

All data used in this study are available in the Supplementary Information and have been deposited in Stockholm University’s Bolin Centre Database (62). The MATLAB code used for statistical source apportionment is available at https://git.bolin.su.se/bolin/wild-2019 (63).

## Supplementary Material

Supplementary File
