# Supplementary material for: Rivers across the Siberian Arctic unearth the patterns of carbon release from thawing permafrost
Source: Proc Natl Acad Sci U S A. 2019 May 6;116(21):10280–5. doi: 10.1073/pnas.1811797116 (PMC6535028; doi:10.1073/pnas.1811797116)
Supplement: Supplementary File [file pnas.1811797116.sapp.pdf]

PNAS
www.pnas.org

Supplementary Information for

Rivers across the Siberian Arctic unearth the patterns of carbon release from thawing permafrost

Birgit Wild, August Andersson, Lisa Bröder, Jorien Vonk, Gustaf Hugelius, James W. McClelland, Wenjun Song, Peter A. Raymond, Örjan Gustafsson

Birgit Wild
Email: birgit.wild@aces.su.se

Örjan Gustafsson
Email: orjan.gustafsson@aces.su.se

This PDF file includes:

Supplementary text
Figs. S1 to S4
Tables S1 to S9
References for SI reference citations

www.pnas.org/cgi/doi/10.1073/pnas.1811797116

Supplementary Information Text

Sampling and analyses of DOC and POC

Samples for DOC and POC analyses were collected at Salekhard (Ob), Dudinka (Yenisey), Zhigansk (Lena) and Cherskiy (Kolyma) between July 2003 and November 2013 as part of the PARTNERS and ARCTIC-GRO programs (1). Water was collected with US Geological Survey D-96 depth-integrating water samplers from 2003 through 2011, and with Van Dorn bottles deployed at discrete water depths starting in 2012 during ice-free conditions. In both cases, multiple casts across the river channel were combined in a Teflon churn to create composite samples for analysis. During ice-covered periods, surface water samples were collected through holes cut into the ice. Water for ^{13}C and ^{14}C analyses of DOC was filtered through Whatman QM-A filters (2.2 µm pore size) and stored frozen in acid-leached polycarbonate bottles until analysis. DOC samples were UV oxidized and cryogenically purified before ^{13}C and ^{14}C analysis at the US-NSF National Ocean Sciences Accelerator Mass Spectrometry (NOSAMS) facility of the Woods Hole Oceanographic Institution (Woods Hole, Massachusetts, USA). For ^{13}C analyses of POC, water was filtered through pre-combusted Whatman GF/F filters (0.7 µm pore size). Filters were frozen and transported to the Marine Biological Laboratory in Woods Hole, where they were dried at 60°C, triple acidified with sulfurous acid (H_2SO_3) to remove inorganic carbon, re-dried, and packed into tin capsules. The $\delta^{13}C$ values of POC were analyzed with Elemental Analysis – Isotope Ratio Mass Spectrometry at the Marine Biological Laboratory between 2003 and 2011 and at the University of Texas Marine Science Institute thereafter. For ^{14}C analysis of POC, water was filtered through pre-combusted Whatman QM-A quartz filters (2.2 µm pore size) that were then acidified with H_2SO_3 and analyzed for ^{14}C at the NOSAMS facility. Details on DOC and POC sampling and analysis methods can be found in previously published papers (2–4), as well as in the metadata provided with the publicly available datasets (www.arcticgreatrivers.org).

Main sources of organic carbon to Siberian rivers

Overview. We distinguished four potential organic carbon sources for source apportionment, (i) recent terrestrial primary production, (ii) active layers (including non-permafrost surface soils), (iii) Holocene permafrost, peat and thermokarst deposits, and (iv) Pleistocene permafrost deposits such as Ice Complex Deposits. Note that aquatic primary production in high-latitude rivers represents mostly recycling of terrestrially-derived, mineralized carbon and is not considered an independent carbon source (see main text for discussion). The isotopic composition of potential carbon sources was constrained based on meta-analyses of previous publications, focusing on Siberia, but extending the spatial coverage where data from Siberia were scarce. Conventional ^{14}C ages (before 1950) reported in the literature were converted into $\Delta^{14}C$ values. The $\Delta^{14}C$ values of organic carbon sources were used for quantitative statistical source apportionment, and the $\delta^{13}C$ values for qualitative comparison to assess the degree of processing of organic carbon in Siberian rivers. Individual carbon sources, and their $\Delta^{14}C$ and $\delta^{13}C$ values are briefly summarized in Table S1, and in more detail below.

Terrestrial primary production. We estimated the $\Delta^{14}C$ and $\delta^{13}C$ values of carbon recently fixed by terrestrial plants from measurements of litter and surface organic layers in arctic, subarctic, and boreal systems. Since data from Siberia were scarce (n = 1 for $\Delta^{14}C$, n = 10 for $\delta^{13}C$; see Table S2), we included also observations from the European part of northern Russia, northern Scandinavia, northern Canada, and Alaska. We followed the description of the original authors for identifying litter and surface organic layers; data for mineral surface soils from Siberia are included in the active layer category. Data and references are presented in Table S2. Note that for most samples, only $\delta^{13}C$ or $\Delta^{14}C$ values are reported. The $\delta^{13}C$ values of organic and litter layers were thus constrained as -27.7 ± 1.3‰ (n = 94) and the $\Delta^{14}C$ values as 97.0 ± 124.8‰ (n =

58), indicating the dominance of carbon taken up between the 1950s and the present, when atmospheric CO_2 was enriched in ^{14}C due to nuclear weapons tests. The decadal ^{14}C age of terrestrial primary production (indicated by the enriched "bomb spike" ^{14}C signature (5)), reflects the residence time of terrestrial plant bio- and necromass in the system.

Active layer. Active layer $\Delta^{14}C$ and $\delta^{13}C$ values were estimated from measurements of the seasonally thawed part of permafrost soils, as well as of non-permafrost soils in Siberia. We thus also consider permafrost-free landscapes in the south of the Ob and Yenisey catchments. All samples were from depths of less than 1 m. Samples that were described as surface organic layers by the original authors are not included here, but in the terrestrial primary production category. We thus estimated $\delta^{13}C$ values of -26.4 ± 0.8‰ (n = 56) and $\Delta^{14}C$ values of -197.5 ± 148.3‰ (n = 60) for active layers. Data and references are presented in Table S3.

Holocene permafrost, peat and thermokarst deposits. Average $\Delta^{14}C$ values of Holocene deposits were estimated based on measurements of Holocene peat and thermokarst deposits in Siberia. We restricted our database to samples taken from exposures along river banks and coastlines in order to capture the $\Delta^{14}C$ range of material that may be realistically released into aquatic systems. The final dataset consisted of 138 $\Delta^{14}C$ values from Holocene peat and thermokarst deposits across Siberia that averaged -567.5 ± 156.7‰ (Table S4). Reports of $\delta^{13}C$ values are scarce for peat deposits in Siberia, but measurements of peat cores in Finland, Sweden, Canada, and the European part of Russia fall mostly between -25 and -28‰ (6–13). For Holocene thermokarst deposits, organic matter $\delta^{13}C$ values between -25 and -29.5‰ have been observed in north-eastern Siberia (14), and between -23 and -28.5‰ in the Alaskan and Canadian Arctic (15–18). This overall range is characteristic for terrestrial organic matter in systems dominated by C3 plants, and closely matches the ranges for terrestrial primary production (-27.7 ± 1.3, n = 94; Table S2), active layers (-26.4 ± 0.8‰, n = 56; Table S3) and Pleistocene deposits (-26.3 ± 0.7‰; Table S5) used in this study.

Pleistocene deposits. We constrained $\Delta^{14}C$ and $\delta^{13}C$ values of Pleistocene deposits using observations from Pleistocene Ice Complex Deposits (also known as "Yedoma") that are most vulnerable to degradation due to their high ice content. The $\delta^{13}C$ values were estimated based on a previous review (19) as -26.3 ± 0.7‰ (n = 374), and $\Delta^{14}C$ values by updating the database used by Vonk et al. (20) and Tesi et al. (21). This new database includes a set of more recent publications, but excludes samples where contamination by Holocene material was indicated (see also ref. (21)). As for Holocene deposits, we restricted our database to observations from river bank and coastal exposures in order to capture the $\Delta^{14}C$ range of material realistically remobilized to aquatic systems. Only data from Siberia were considered. The final database is presented in Table S5, and $\Delta^{14}C$ values were estimated as -954.8 ± 65.8‰ (n = 329).

Source apportionment

Fractions of organic carbon from recent primary production as opposed to permafrost and peat deposits (PP-C) in river samples were calculated using Equations (1) and (2), based on the $\Delta^{14}C$ values of samples ($\Delta^{14}C_{sample}$), recent primary production ($\Delta^{14}C_{recent}$) and PP-C ($\Delta^{14}C_{PP}$). f_{recent} and f_{PP} are the fractions of recent carbon and PP-C, respectively.

$$1 = f_{recent} + f_{PP} \quad (1)$$

$$\Delta^{14}C_{sample} = f_{PP} \cdot \Delta^{14}C_{PP} + (1 - f_{PP}) \cdot \Delta^{14}C_{recent} \quad (2)$$

The $\Delta^{14}C$ values of the PP-C endmember were calculated for three scenarios, assuming different contributions of organic carbon from active layer, Holocene deposits and Pleistocene deposits.

The Best Estimate scenario represents, in our opinion, the most realistic estimate as it assumes that all PP-C compartments contribute to fluvial PP-C. A least biased approach was used where all fractional combinations of individual compartments are set to be equally likely. Formally, we thus integrated over all possible fractional combinations of the individual compartment distributions to compute the Best Estimate distribution. Compared to the assumption of equal contribution of all compartments, this approach results in a wider spread of the combined PP-C probability density function compared to the assumption of a fixed mixing ratio, and a larger uncertainty that considers not only the uncertainties of the $\Delta^{14}C$ values of individual compartments but also the uncertainty of their relative proportions, and is therefore more conservative. Active layer as well as Holocene deposits were considered for all rivers, whereas Pleistocene deposits were only considered for Lena and Kolyma catchments where they are abundant. Sensitivity to the Best Estimate assumptions was tested in the Maximum and Minimum scenarios that assume a contribution of only the youngest (Maximum) or oldest (Minimum) PP-C compartment to fluvial PP-C. In the Maximum scenario, the PP-C endmember was thus set to the active layer values, and in the Minimum scenario, to the values of Holocene deposits (Ob and Yenisey) or Pleistocene deposits (Lena and Kolyma). Maximum and Minimum scenarios constrain the upper and lower limit of organic carbon from permafrost and peat deposits in Ob, Yenisey, Lena, and Kolyma.

To account for the uncertainties of the $\Delta^{14}C$ endmember distribution, a Bayesian approach was implemented. A uniform ("objective") prior distribution was used to represent our initial knowledge of the fractional source contributions. The prior then was combined with the knowledge from the measurements assuming isotopic mass-balance ("the likelihood") to compute the posterior distribution of the fractional contributions, from which statistical parameters such as mean, median and standard deviations can be derived. Here, the method presented in Andersson et al. (22) was adjusted to accommodate flux weighting, with fluxes calculated from discharge and POC or DOC concentration at the respective time point. The weight from data point i (w_i) was computed

$$w_i = \frac{J_i}{\sum_{i=1}^{N} J_i} \tag{3}$$

where J is the flux and N the number of observations. The posterior distribution of the fractional source contribution can then be expressed as:

$$P(f|\Delta^{14}C) = \left(\prod_{i=1}^{N} p(\Delta^{14}C_i|f)^{N\cdot w_i}\right)\cdot p(f) \tag{4}$$

where p(f) is the prior and $\prod_{i=1}^{N} p(\Delta^{14}C_i|f)^{N\cdot w_i}$ the likelihood. A Markov chain Monte Carlo (MCMC) algorithm (22) implemented in Matlab (ver. 2014b) was used to compute the posterior. To ensure good convergence and a low computation uncertainty, the calculations were run using 1 000 000 iterations, a burn-in of 10 000 and a data thinning of 10.

Response of fluvial $\Delta^{14}C$ values to changes in PP-C release

An additional simulation was performed to test the sensitivity of fluvial organic carbon $\Delta^{14}C$ values to changes in PP-C release. To that end, the flux of PP-C in rivers was changed by a factor x ranging from 0.5 to 2.0 in 0.25 increments, while recent carbon flux was kept constant. The thus adjusted PP-C fraction of total fluvial carbon ($f_{PP\text{-}change}$) and the resulting shift in $\Delta^{14}C$ values of fluvial organic carbon were calculated using Equations (5) and (6).

$$f_{PP-change} = \frac{x\cdot f_{PP}}{x\cdot f_{PP}+(1-f_{PP})} \tag{5}$$

$$\Delta^{14}C\ shift = \left(f_{PP-change} - f_{PP}\right) \cdot \Delta^{14}C_{PP} + \left(f_{PP} - f_{PP-change}\right) \cdot \Delta^{14}C_{recent} \qquad (6)$$

The minimum resolvable change in PP-C flux was calculated for each river by changing measured $\Delta^{14}C$ values in 1‰ increments and comparing the resulting dataset with baseline values using flux-weighted t-tests. The minimum change in $\Delta^{14}C$ that resulted in a statistically significant difference ($p < 0.05$) was then inserted into Equations (5) and (6) to derive the corresponding factor x. Calculations were performed in R 3.5.1 (23) with the packages 'Hmisc' (24) and 'weights' (25).

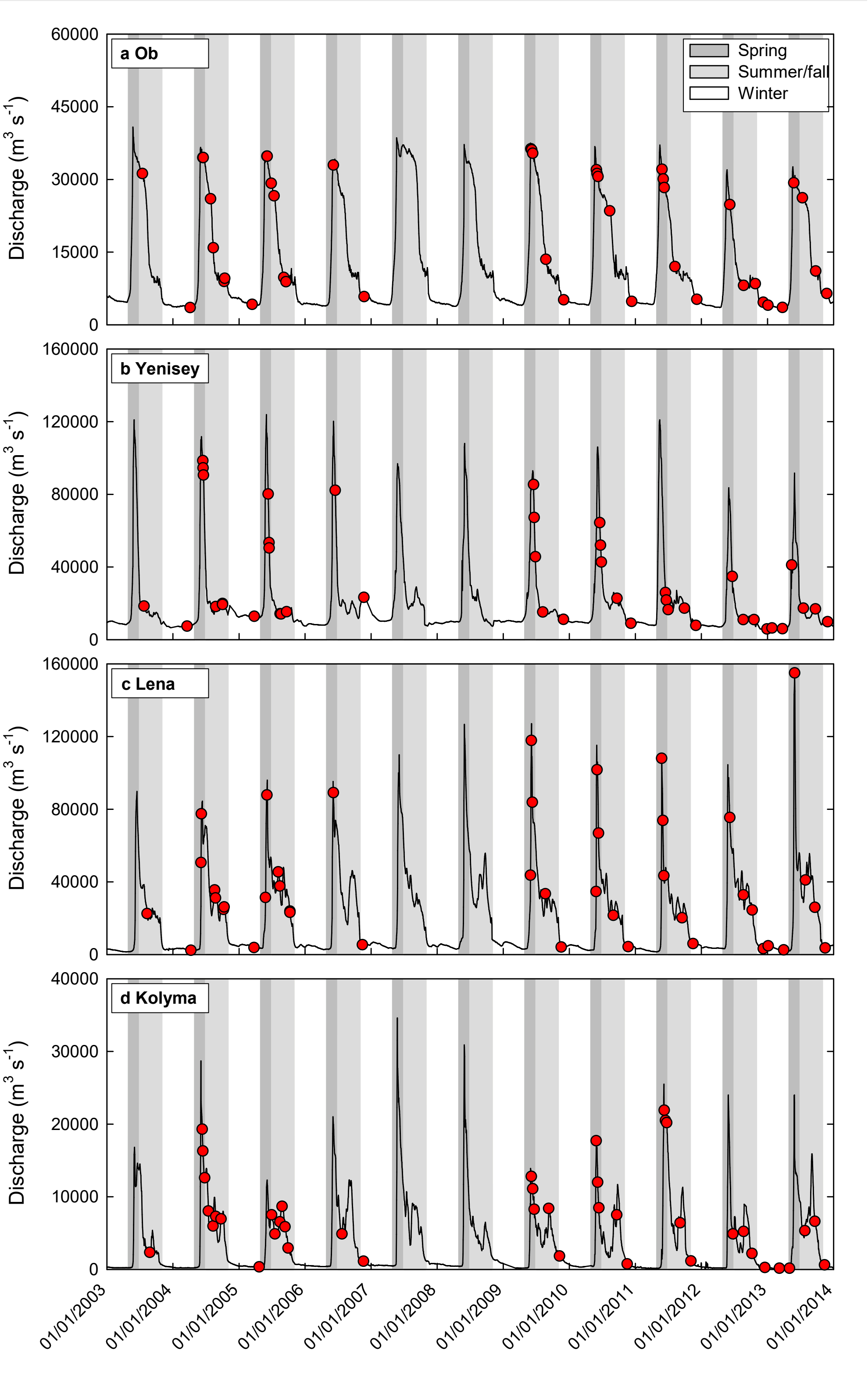

Fig. S1. Discharge over time for Ob, Yenisey, Lena and Kolyma, with spring, summer/fall and winter seasons marked in different shades of grey. Red circles indicate sampling dates for POC and DOC analyses. See Table S6 for further details.

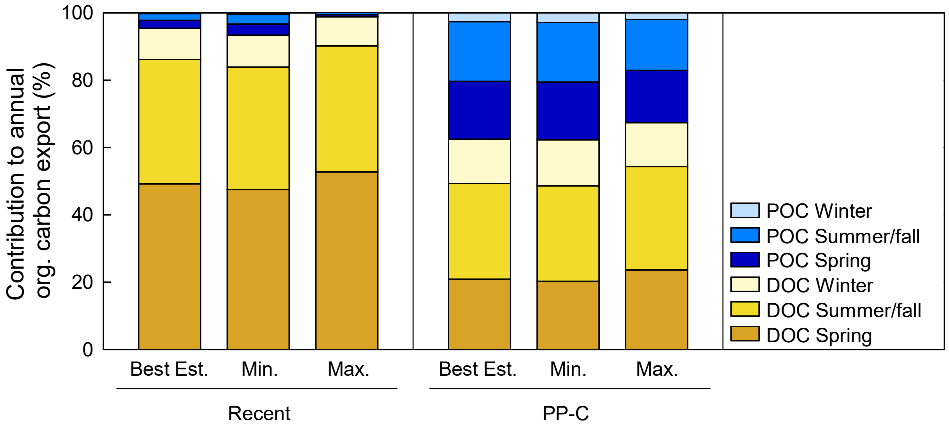

Fig. S2. Contribution of DOC and POC in spring, summer/fall and winter to the annual export of recent organic carbon as well as permafrost and peat carbon (PP-C), calculated for the Best Estimate, Minimum, and Maximum scenarios. Total organic carbon export was quantified based on discharge, POC and DOC concentration measurements using the LOADEST program (2, 3), and recent carbon vs PP-C were dissected using ^{14}C-based source apportionment.

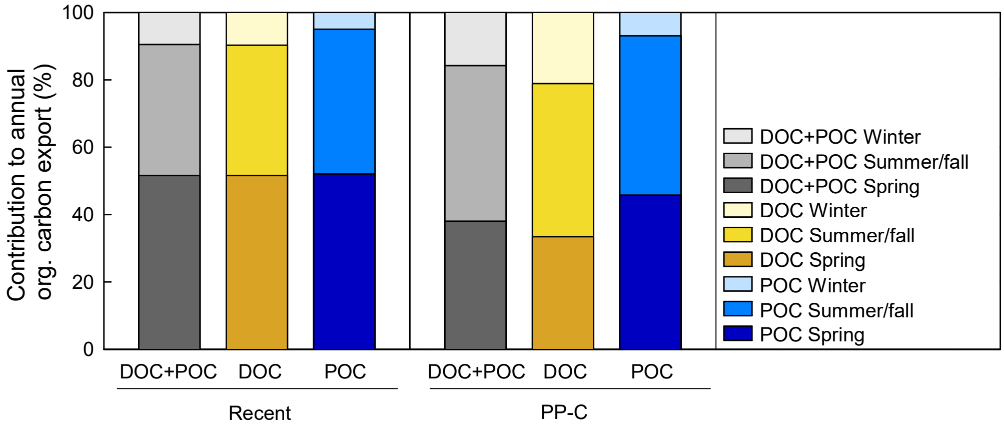

Fig. S3. Contribution of spring, summer/fall and winter to the annual export of recent organic carbon as well as permafrost and peat carbon (PP-C) in dissolved (DOC) and particulate (POC) form (Best Estimate scenario). Total organic carbon export was quantified based on discharge, POC and DOC concentration measurements using the LOADEST program (2, 3), and recent carbon vs PP-C were dissected using ^{14}C-based source apportionment.

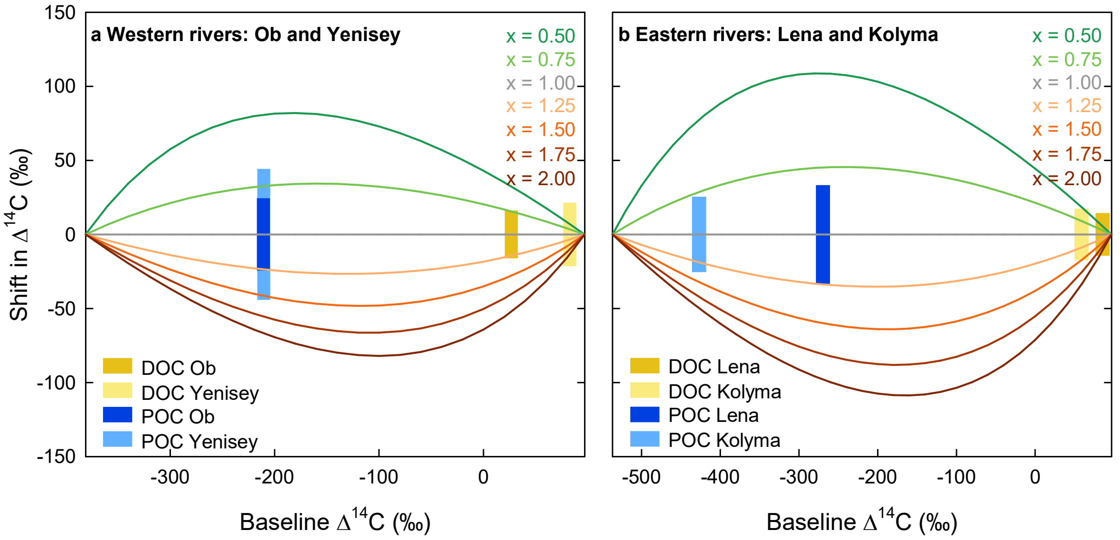

Fig. S4. Simulated response of fluvial organic carbon $\Delta^{14}C$ values to changes in PP-C flux by a factor of x, depending on baseline $\Delta^{14}C$ values (i.e., without PP-C change). Flux-weighted, average $\Delta^{14}C$ values of DOC and POC in individual rivers are indicated by bars, with the height of each bar corresponding to the minimum change in $\Delta^{14}C$ that would be resolved as statistically different compared to the baseline value ($p < 0.05$).

Table S1. Overview of $\Delta^{14}C$ and $\delta^{13}C$ values of potential organic carbon sources.

Table S1. Overview of $\Delta^{14}C$ and $\delta^{13}C$ values of potential organic carbon sources. Sd, standard deviation; n, number of observations. Details on data sources are provided in the Supplementary Information Text.

Carbon source	Mean ±	Sd	n	Data source
$\Delta^{14}C$ (‰)				
Terrestrial primary production	97.0 ±	124.8	58	Org. and litter layers in n. Russia, Scandinavia, Canada, Alaska
Active layer	-197.5 ±	148.3	60	Min. active layers and non-permafrost soils in Siberia
Holocene deposits	-567.5 ±	156.7	138	Exposures of Holocene peat and thermokarst deposits in Siberia
Pleistocene deposits	-954.8 ±	65.8	329	Exposures of Pleistocene Ice Complex Deposits in Siberia
$\delta^{13}C$ (‰)				
Terrestrial primary production	-27.7 ±	1.3	94	Org. and litter layers in n. Russia, Scandinavia, Canada, Alaska
Active layer	-26.4 ±	0.8	56	Min. active layers and non-permafrost soils in Siberia
Holocene deposits	n.a.			
Pleistocene deposits	-26.3 ±	0.7	374	Pleistocene Ice Complex Deposits in Siberia

Table S2. Database of $\delta^{13}C$ and $\Delta^{14}C$ values of organic layer and litter samples.

Table S2. Database of $\delta^{13}C$ and $\Delta^{14}C$ values of organic layer (Org.) and litter samples from Northern Russia (including Siberia), Northern Scandinavia, Northern Canada, and Alaska. Lat., latitude; Long., longitude; repl., replicates.

Location	Lat.	Long.	Type	$\delta^{13}C$ (‰)	$\Delta^{14}C$ (‰)	^{14}C Lab ID	Reference
Newfoundland, Canada	51.2°N	56.0°W	Org.	-28.6	108.0		(26)
Newfoundland, Canada	51.2°N	56.0°W	Org.	-26.8	207.7		(26)
Newfoundland, Canada	47.5°N	59.2°W	Org.	-29.5	120.5		(26)
Newfoundland, Canada	47.5°N	59.2°W	Org.	-28.9	125.4		(26)
Sagwon Hills, Alaska	69.4°N	148.7°W	Org.	-26.4	-20.0		(27)
Eastern Siberia	69.2°N	161.7°E	Org.	-28.3			(28) Mean of 15 repl.
Taymyr Peninsula, Siberia	72.5°N	101.7°E	Org.	-27.9			(28) Mean of 3 repl.
Taymyr Peninsula, Siberia	73.4°N	98.4°E	Org.	-28.4			(28) Mean of 6 repl.
Western Siberia	67.2°N	78.9°E	Org.	-26.6			(28) Mean of 7 repl.
Abisko, northern Sweden	68.4°N	18.8°E	Org.	-27.5			(29)
Western Siberia	67.3°N	78.8°E	Org.	-26.6			(30) Mean of 5 repl.
Western Siberia	63.3°N	74.5°E	Org.	-28.3			(30) Mean of 5 repl.
Western Siberia	60.3°N	71.7°E	Org.	-29.0			(30) Mean of 5 repl.
Western Siberia	58.3°N	68.6°E	Org.	-28.6			(30) Mean of 5 repl.
Western Siberia	56.2°N	70.7°E	Org.	-27.9			(30) Mean of 5 repl.
Western Siberia	56.2°N	70.7°E	Org.	-27.6			(30) Mean of 5 repl.
Northern Sweden	66.0°N	17.8°E	Org.		150.0		(31) Mean of 3 repl.
Northern Sweden	66.0°N	17.8°E	Org.		21.0		(31) Mean of 3 repl.
Northern Sweden	66.0°N	17.8°E	Org.		9.4		(31) Mean of 3 repl.
Northern Sweden	66.0°N	17.8°E	Org.		191.0		(31) Mean of 3 repl.
Northern Sweden	66.0°N	17.8°E	Org.		195.0		(31) Mean of 3 repl.
Northern Sweden	66.0°N	17.8°E	Org.		14.0		(31) Mean of 3 repl.
Northern Sweden	66.0°N	17.8°E	Org.		-99.0		(31) Mean of 3 repl.
Northern Sweden	66.0°N	17.8°E	Org.		-194.0		(31) Mean of 3 repl.
Northern Sweden	66.0°N	17.8°E	Org.		-285.0		(31) Mean of 3 repl.
Northern Sweden	66.0°N	17.8°E	Org.		-267.0		(31) Mean of 3 repl.
Usa Basin, European Russia	67.1°N	62.9°E	Org.		115.3	Poz-35117	(32)
Usa Basin, European Russia	67.1°N	62.9°E	Org.		23.5	Poz-35119	(32)
Usa Basin, European Russia	67.1°N	62.9°E	Org.		75.9	Poz-35120	(32)
Gydansky Peninsula, Siberia	69.7°N	74.6°E	Org.		130.1		(33)
Toolik Lake, Alaska	68.6°N	149.6°W	Org.		177.3		(34)
Toolik Lake, Alaska	68.6°N	149.6°W	Org.		98.0		(34)
Abisko, northern Sweden	68.3°N	18.9°E	Org.		263.6	SUERC-12116	(35)
Abisko, northern Sweden	68.3°N	18.9°E	Org.		138.0	SUERC-13099	(35)
Abisko, northern Sweden	68.3°N	18.9°E	Org.		68.5	SUERC-13100	(35)
Abisko, northern Sweden	68.3°N	18.9°E	Org.		27.2	SUERC-12117	(35)
Abisko, northern Sweden	68.3°N	18.9°E	Org.		-30.0	SUERC-12118	(35)
Abisko, northern Sweden	68.3°N	18.9°E	Org.		343.1	SUERC-12126	(35)
Abisko, northern Sweden	68.3°N	18.9°E	Org.		110.3	SUERC-12127	(35)
Central Alaska	65.2°N	147.5°W	Litter	-29.3	182.4		(36)
Central Alaska	65.2°N	147.5°W	Litter	-27.1	253.7		(36)
Central Alaska	65.2°N	147.5°W	Litter	-25.3	340.8		(36)
Western Russia	56.0°N	38.4°E	Litter	-28.8			(37)
Tanana floodplain, Alaska	64.9°N	147.7°W	Litter	-27.6			(38)
Tanana floodplain, Alaska	64.9°N	147.7°W	Litter	-27.2			(38)
Tanana floodplain, Alaska	64.9°N	147.7°W	Litter	-28.5			(38)
Abisko, northern Sweden	68.4°N	18.8°E	Litter	-31.8			(29)
Northwestern Russia	61.6°N	50.7°E	Litter	-28.9			(39)
Northwestern Russia	61.6°N	50.7°E	Litter	-32.4			(39)

Northwestern Russia	61.6°N	50.7°E	Litter	-32.3	(39)
Abisko, northern Sweden	68.3°N	19.1°E	Litter	-27.1	(10)
Abisko, northern Sweden	68.3°N	19.1°E	Litter	-28.3	(10)
Abisko, northern Sweden	68.3°N	19.1°E	Litter	-28.4	(10)
Alberta, Canada	57.0°N	111.6°W	Litter	-29.2	(40)
Alberta, Canada	56.9°N	111.7°W	Litter	-28.5	(40)
Alberta, Canada	57.0°N	111.6°W	Litter	-28.0	(40)
Alberta, Canada	56.9°N	111.7°W	Litter	-28.1	(40)
Saskatchewan, Canada	53.2°N	105.8°W	Litter	-28.7	(41)
Saskatchewan, Canada	53.2°N	105.8°W	Litter	-27.7	(41)
Manitoba, Canada	55.8°N	98.0°W	Litter	-27.3	(41)
Manitoba, Canada	55.8°N	98.0°W	Litter	-27.5	(41)
Manitoba, Canada	56.5°N	94.9°W	Litter	-27.5	(41)
Saskatchewan, Canada	53.2°N	105.8°W	Litter	-26.2	(41)
Saskatchewan, Canada	53.2°N	105.8°W	Litter	-26.4	(41)
Saskatchewan, Canada	53.2°N	105.8°W	Litter	-28.8	(41)
Saskatchewan, Canada	53.2°N	105.8°W	Litter	-27.7	(41)
Manitoba, Canada	55.8°N	98.0°W	Litter	-26.3	(41)
Manitoba, Canada	55.8°N	98.0°W	Litter	-27.2	(41)
Manitoba, Canada	55.8°N	98.0°W	Litter	-26.4	(41)
Manitoba, Canada	55.8°N	98.0°W	Litter	-26.2	(41)
Manitoba, Canada	55.8°N	98.0°W	Litter	-27.1	(41)
Manitoba, Canada	55.8°N	98.0°W	Litter	-26.5	(41)
Manitoba, Canada	55.8°N	98.0°W	Litter	-25.6	(41)
Manitoba, Canada	56.5°N	94.9°W	Litter	-27.3	(41)
Manitoba, Canada	56.5°N	94.9°W	Litter	-26.4	(41)
Manitoba, Canada	56.5°N	94.9°W	Litter	-25.9	(41)
Saskatchewan, Canada	53.2°N	105.8°W	Litter	-28.5	(41)
Saskatchewan, Canada	53.2°N	105.8°W	Litter	-27.9	(41)
Saskatchewan, Canada	54.8°N	101.9°W	Litter	-28.0	(41)
Manitoba, Canada	55.8°N	98.0°W	Litter	-28.2	(41)
Manitoba, Canada	55.8°N	98.0°W	Litter	-28.1	(41)
Manitoba, Canada	56.5°N	94.9°W	Litter	-28.0	(41)
Saskatchewan, Canada	53.2°N	105.8°W	Litter	-28.2	(41)
Saskatchewan, Canada	53.2°N	105.8°W	Litter	-27.1	(41)
Saskatchewan, Canada	54.8°N	101.9°W	Litter	-27.5	(41)
Manitoba, Canada	55.8°N	98.0°W	Litter	-27.6	(41)
Manitoba, Canada	55.8°N	98.0°W	Litter	-27.7	(41)
Manitoba, Canada	56.5°N	94.9°W	Litter	-28.3	(41)
Saskatchewan, Canada	53.2°N	105.8°W	Litter	-28.4	(41)
Saskatchewan, Canada	53.2°N	105.8°W	Litter	-27.4	(41)
Saskatchewan, Canada	54.8°N	101.9°W	Litter	-25.5	(41)
Manitoba, Canada	55.8°N	98.0°W	Litter	-27.5	(41)
Manitoba, Canada	55.8°N	98.0°W	Litter	-26.5	(41)
Manitoba, Canada	56.5°N	94.9°W	Litter	-27.9	(41)
Saskatchewan, Canada	54.8°N	101.9°W	Litter	-29.5	(41)
Manitoba, Canada	55.8°N	98.0°W	Litter	-28.4	(41)
Manitoba, Canada	56.5°N	94.9°W	Litter	-29.4	(41)
Saskatchewan, Canada	53.2°N	105.8°W	Litter	-27.3	(41)
Saskatchewan, Canada	53.2°N	105.8°W	Litter	-28.0	(41)
Saskatchewan, Canada	54.8°N	101.9°W	Litter	-27.2	(41)
Manitoba, Canada	55.8°N	98.0°W	Litter	-27.3	(41)
Manitoba, Canada	55.8°N	98.0°W	Litter	-27.2	(41)
Manitoba, Canada	56.5°N	94.9°W	Litter	-27.9	(41)
Saskatchewan, Canada	53.2°N	105.8°W	Litter	-26.9	(41)
Saskatchewan, Canada	53.2°N	105.8°W	Litter	-26.5	(41)

Saskatchewan, Canada	54.8°N	101.9°W	Litter	-25.8		(41)	
Manitoba, Canada	55.8°N	98.0°W	Litter	-26.2		(41)	
Manitoba, Canada	55.8°N	98.0°W	Litter	-26.0		(41)	
Manitoba, Canada	56.5°N	94.9°W	Litter	-26.5		(41)	
Saskatchewan, Canada	53.2°N	105.8°W	Litter	-26.2		(41)	
Saskatchewan, Canada	53.2°N	105.8°W	Litter	-26.2		(41)	
Saskatchewan, Canada	54.8°N	101.9°W	Litter	-25.2		(41)	
Manitoba, Canada	55.8°N	98.0°W	Litter	-26.2		(41)	
Manitoba, Canada	55.8°N	98.0°W	Litter	-26.3		(41)	
Manitoba, Canada	56.5°N	94.9°W	Litter	-26.2		(41)	
Saskatchewan, Canada	54.8°N	101.9°W	Litter	-29.2		(41)	
Saskatchewan, Canada	53.2°N	105.8°W	Litter	-29.3		(41)	
Northern Sweden	66.0°N	17.8°E	Litter		70.0	(31)	Mean of 3 repl.
Northern Sweden	66.0°N	17.8°E	Litter		81.0	(31)	Mean of 3 repl.
Northern Sweden	66.0°N	17.8°E	Litter		118.0	(31)	Mean of 3 repl.
Northern Sweden	66.0°N	17.8°E	Litter		165.0	(31)	Mean of 3 repl.
Northern Sweden	66.0°N	17.8°E	Litter		62.0	(31)	Mean of 3 repl.
Northern Sweden	66.0°N	17.8°E	Litter		84.0	(31)	Mean of 3 repl.
Northern Sweden	66.0°N	17.8°E	Litter		101.0	(31)	Mean of 3 repl.
Northern Sweden	66.0°N	17.8°E	Litter		136.0	(31)	Mean of 3 repl.
Manitoba, Canada	55.9°N	98.2°W	Litter		57.7	(42)	
Manitoba, Canada	55.9°N	98.2°W	Litter		255.8	(42)	
Manitoba, Canada	56.6°N	99.9°W	Litter		-11.5	(42)	
Manitoba, Canada	56.6°N	99.9°W	Litter		71.2	(42)	
Manitoba, Canada	56.6°N	99.9°W	Litter		117.3	(42)	
Manitoba, Canada	56.6°N	99.9°W	Litter		246.2	(42)	
Manitoba, Canada	55.9°N	99.0°W	Litter		9.6	(42)	
Manitoba, Canada	55.9°N	99.0°W	Litter		100.0	(42)	
Manitoba, Canada	55.9°N	98.4°W	Litter		1.9	(42)	
Manitoba, Canada	55.9°N	98.4°W	Litter		65.4	(42)	
Manitoba, Canada	55.9°N	98.4°W	Litter		142.3	(42)	
Manitoba, Canada	55.9°N	98.5°W	Litter		103.8	(42)	
Manitoba, Canada	55.9°N	98.5°W	Litter		176.9	(42)	
Manitoba, Canada	55.9°N	98.5°W	Litter		282.7	(42)	
Manitoba, Canada	55.9°N	98.5°W	Litter		315.4	(42)	
Manitoba, Canada	55.9°N	98.5°W	Litter		-13.5	(42)	
Manitoba, Canada	55.9°N	98.5°W	Litter		23.1	(42)	
Manitoba, Canada	55.9°N	98.5°W	Litter		130.8	(42)	
Toolik Lake, Alaska	68.6°N	149.6°W	Litter		138.4	(34)	
Mean (Org.)				-27.9	64.9		
Standard deviation (Org.)				0.9	145.0		
Number of observations (Org.)				16	28		
Mean (Litter)				-27.6	126.9		
Standard deviation (Litter)				1.4	95.4		
Number of observations (Litter)				78	30		
Mean (all)				-27.7	97.0		
Standard deviation (all)				1.3	124.8		
Number of observations (all)				94	58		

Table S3. Database of $\delta^{13}C$ and $\Delta^{14}C$ values of mineral active layer and non-permafrost soils.

Table S3. Database of $\delta^{13}C$ and $\Delta^{14}C$ values measured in the mineral active layer (AL) and mineral non-permafrost soils (NPF) in Siberia. Lat., latitude; Long., longitude; repl., replicates.

Location	Lat.	Long.	Type	$\delta^{13}C$ (‰)	$\Delta^{14}C$ (‰)	^{14}C Lab ID	Reference
Eastern Siberia	68.8°N	161.4°E	AL	-25.3	-421.0		(43)
Eastern Siberia	69.2°N	161.7°E	AL	-26.9			(28) Mean of 12 repl.
Eastern Siberia	69.2°N	161.7°E	AL	-27.5			(28) Mean of 26 repl.
Eastern Siberia	69.2°N	161.7°E	AL	-26.4			(28) Mean of 25 repl.
Taymyr Peninsula, Siberia	72.5°N	101.7°E	AL	-27.5			(28) Mean of 11 repl.
Taymyr Peninsula, Siberia	72.5°N	101.7°E	AL	-27.2			(28) Mean of 38 repl.
Taymyr Peninsula, Siberia	72.5°N	101.7°E	AL	-26.2			(28) Mean of 17 repl.
Taymyr Peninsula, Siberia	73.4°N	98.4°E	AL	-27.9			(28) Mean of 10 repl.
Taymyr Peninsula, Siberia	73.4°N	98.4°E	AL	-27.4			(28) Mean of 25 repl.
Taymyr Peninsula, Siberia	73.4°N	98.4°E	AL	-26.4			(28) Mean of 19 repl.
Western Siberia	67.2°N	78.9°E	AL	-25.8			(28) Mean of 6 repl.
Western Siberia	67.2°N	78.9°E	AL	-25.8			(28) Mean of 18 repl.
Western Siberia	67.2°N	78.9°E	AL	-24.1			(28) Mean of 24 repl.
Taymyr Peninsula, Siberia	72.0°N	99.0°E	AL	-26.6			(44)
Taymyr Peninsula, Siberia	72.0°N	99.0°E	AL	-25.8			(44)
Taymyr Peninsula, Siberia	72.0°N	99.0°E	AL	-26.3			(44)
Taymyr Peninsula, Siberia	72.0°N	99.0°E	AL	-26.4			(44)
Central Siberia	67.5°N	86.4°E	AL	-27.2			(45)
Central Siberia	67.5°N	86.4°E	AL	-25.6			(45)
Central Siberia	67.5°N	86.4°E	AL	-27.0			(45)
Central Siberia	67.5°N	86.4°E	AL	-26.8			(45)
Western Siberia	67.3°N	78.8°E	AL	-25.7			(30) Mean of 5 repl.
Western Siberia	67.3°N	78.8°E	AL	-24.7			(30) Mean of 5 repl.
Lena Delta, Siberia	72.4°N	126.5°E	AL		-106.5		(46)
Lena Delta, Siberia	72.4 N	126.5°E	AL		-176.0		(46)
Lena Delta, Siberia	72.4°N	126.5°E	AL		-225.6		(46)
Eastern Siberia	69.4°N	125.1°E	AL		-303.6	WAT-2893	(47)
Eastern Siberia	69.4°N	125.1°E	AL		-399.5	WAT-3007	(47)
Gydansky Peninsula, Siberia	69.7°N	74.6°E	AL		-50.0		(33)
Gydansky Peninsula, Siberia	69.7°N	74.6°E	AL		-377.3		(33)
Gydansky Peninsula, Siberia	69.7°N	74.6°E	AL		-165.7		(33)
Gydansky Peninsula, Siberia	69.7°N	74.6°E	AL		-539.9		(33)
Gydansky Peninsula, Siberia	69.7°N	74.6°E	AL		-148.8		(33)
Eastern Siberia	69.5°N	161.8°E	AL		-46.6	Poz-38344	(48)
Eastern Siberia	69.5°N	161.8°E	AL		-246.0	Poz-39963	(48)
Eastern Siberia	69.5°N	161.8°E	AL		-134.8	Poz-38341	(48)
Eastern Siberia	69.5°N	161.8°E	AL		-182.0	Poz-38343	(48)
Eastern Siberia	69.5°N	161.8°E	AL		-221.2	Poz-38339	(48)
Eastern Siberia	69.5°N	161.8°E	AL		-227.0	Poz-38342	(48)
Eastern Siberia	69.5°N	161.8°E	AL		-344.5	Poz-39960	(48)
Eastern Siberia	69.5°N	161.8°E	AL		-330.9	Poz-38334	(48)
Eastern Siberia	69.5°N	161.8°E	AL		-45.4	Poz-38338	(48)
Eastern Siberia	69.5°N	161.8°E	AL		-96.3	Poz-38346	(48)
Eastern Siberia	69.5°N	161.8°E	AL		-107.5	Poz-38348	(48)
Eastern Siberia	69.5°N	161.8°E	AL		-18.9	Poz-38353	(48)
Taymyr Peninsula, Siberia	72.5°N	101.9°E	AL		-93.6	Poz-59058	(49)
Taymyr Peninsula, Siberia	72.5°N	101.9°E	AL		-190.7	Poz-59056	(49)
Taymyr Peninsula, Siberia	72.5°N	101.9°E	AL		-559.1	Poz-59060	(49)

Taymyr Peninsula, Siberia	72.5°N	101.9°E	AL		-671.0	Poz-59059	(49)
Taymyr Peninsula, Siberia	73.4°N	98.4°E	AL		-107.6	Poz-59038	(49)
Taymyr Peninsula, Siberia	73.4°N	98.4°E	AL		-43.1	Poz-59050	(49)
Taymyr Peninsula, Siberia	73.4°N	98.4°E	AL		-197.2	Poz-59048	(49)
Taymyr Peninsula, Siberia	73.4°N	98.4°E	AL		-278.7	Poz-59037	(49)
Eastern Siberia	70.8°N	147.5°E	AL		3.3	Poz-53462	(50)
Eastern Siberia	70.8°N	147.5°E	AL		-63.9	Poz-53467	(50)
Eastern Siberia	70.8°N	147.5°E	AL		-143.1	Poz-53470	(50)
Eastern Siberia	62.2°N	129.6°E	AL		-94.3	Poz-53529	(50)
Eastern Siberia	62.2°N	129.6°E	AL		-212.6	Poz-53531	(50)
Eastern Siberia	62.2°N	129.6°E	AL		-194.8	Poz-53530	(50)
Eastern Siberia	70.8°N	147.5°E	AL		-37.9		(51)
Eastern Siberia	70.8°N	147.5°E	AL		-75.5		(51)
Central Siberia	56.4°N	93.0°E	NPF	-26.7			(52)
Central Siberia	56.4°N	93.0°E	NPF	-25.6			(52)
Central Siberia	59.3°N	90.7°E	NPF	-27.0			(52)
Central Siberia	59.3°N	90.7°E	NPF	-25.2			(52)
Central Siberia	59.4°N	90.9°E	NPF	-26.7			(52)
Central Siberia	59.4°N	90.9°E	NPF	-25.4			(52)
Central Siberia	60.8°N	89.4°E	NPF	-26.3			(52)
Central Siberia	60.8°N	89.4°E	NPF	-25.4			(52)
Central Siberia	62.3°N	89.0°E	NPF	-27.3			(52)
Central Siberia	62.3°N	89.0°E	NPF	-26.8			(52)
Central Siberia	62.5°N	89.0°E	NPF	-27.5			(52)
Central Siberia	62.5°N	89.0°E	NPF	-26.5			(52)
Central Siberia	63.1°N	87.5°E	NPF	-26.0			(52)
Central Siberia	63.2°N	87.8°E	NPF	-26.3			(52)
Central Siberia	63.2°N	87.8°E	NPF	-26.2			(52)
Central Siberia	64.4°N	87.6°E	NPF	-28.0			(52)
Central Siberia	64.4°N	87.6°E	NPF	-27.0			(52)
Central Siberia	66.0°N	87.7°E	NPF	-27.7			(52)
Central Siberia	66.0°N	87.7°E	NPF	-26.8			(52)
Central Siberia	66.1°N	87.3°E	NPF	-27.9			(52)
Central Siberia	66.1°N	87.3°E	NPF	-26.8			(52)
Central Siberia	67.4°N	86.5°E	NPF	-26.7			(52)
Central Siberia	67.4°N	86.5°E	NPF	-26.9			(52)
Western Siberia	63.3°N	74.5°E	NPF	-27.1			(30) Mean of 5 repl.
Western Siberia	63.3°N	74.5°E	NPF	-25.9			(30) Mean of 5 repl.
Western Siberia	60.3°N	71.7°E	NPF	-26.9			(30) Mean of 5 repl.
Western Siberia	60.3°N	71.7°E	NPF	-26.5			(30) Mean of 5 repl.
Western Siberia	58.3°N	68.6°E	NPF	-26.8			(30) Mean of 5 repl.
Western Siberia	58.3°N	68.6°E	NPF	-25.3			(30) Mean of 5 repl.
Western Siberia	56.2°N	70.7°E	NPF	-25.7			(30) Mean of 5 repl.
Western Siberia	56.2°N	70.7°E	NPF	-25.4			(30) Mean of 5 repl.
Western Siberia	56.2°N	70.7°E	NPF	-26.2			(30) Mean of 5 repl.
Western Siberia	56.2°N	70.7°E	NPF	-25.9			(30) Mean of 5 repl.
Western Siberia	60.5°N	89.5°E	NPF		-67.1		(53)
Western Siberia	60.5°N	89.5°E	NPF		-250.7		(53)
Western Siberia	60.5°N	89.5°E	NPF		-56.0		(53)
Western Siberia	60.5°N	89.5°E	NPF		-342.1		(53)
Western Siberia	60.5°N	89.5°E	NPF		-72.3		(53)
Western Siberia	60.5°N	89.5°E	NPF		-341.0		(53)
Western Siberia	60.5°N	89.5°E	NPF		-38.2		(53)
Western Siberia	60.5°N	89.5°E	NPF		-66.0		(53)
Western Siberia	60.5°N	89.5°E	NPF		-43.0		(53)
Western Siberia	60.5°N	89.5°E	NPF		-83.8		(53)

Western Siberia	60.5°N	89.5°E	NPF		-27.4	(53)
Western Siberia	60.5°N	89.5°E	NPF		-152.2	(53)
Western Siberia	60.5°N	89.5°E	NPF		-332.3	(53)
Western Siberia	60.5°N	89.5°E	NPF		-152.2	(53)
Western Siberia	60.5°N	89.5°E	NPF		-150.3	(53)
Western Siberia	60.5°N	89.5°E	NPF		-396.2	(53)
Western Siberia	60.5°N	89.5°E	NPF		-342.1	(53)
Western Siberia	60.5°N	89.5°E	NPF		-108.0	(53)
Western Siberia	60.5°N	89.5°E	NPF		-363.8	(53)
Western Siberia	60.5°N	89.5°E	NPF		-244.1	(53)
Western Siberia	60.5°N	89.5°E	NPF		-347.4	(53)
Mean (AL)				-26.4	-201.9	
Standard deviation (AL)				0.9	157.8	
Number of observations (AL)				23	39	
Mean (NPF)				-26.5	-189.3	
Standard deviation (NPF)				0.8	132.1	
Number of observations (NPF)				33	21	
Mean (all)				-26.4	-197.5	
Standard deviation (all)				0.8	148.3	
Number of observations (all)				56	60	

Table S4. Database of $\Delta^{14}C$ values of Holocene peat and thermokarst exposures.

Table S4. Database of $\Delta^{14}C$ values of Holocene peat and thermokarst (THK) deposit exposures in Siberia. Lat., latitude; Long., longitude.

Location	Lat.	Long.	Type	$\Delta^{14}C$ (‰)	^{14}C Lab ID	Reference
Yana-Kolyma Lowland, Siberia	70.8°N	136.3°E	Peat	-63.8	GIN-5383a	(54)
Yana-Kolyma Lowland, Siberia	70.8°N	136.3°E	Peat	-178.5	GIN-5385a	(54)
Yana-Kolyma Lowland, Siberia	70.8°N	136.3°E	Peat	-395.2	GIN-5386	(54)
Yana-Kolyma Lowland, Siberia	70.8°N	136.3°E	Peat	-398.2	GIN-5387a	(54)
Yana-Kolyma Lowland, Siberia	70.8°N	136.3°E	Peat	-415.2	GIN-5387b	(54)
Yana-Kolyma Lowland, Siberia	70.8°N	136.3°E	Peat	-463.3	GIN-5388	(54)
Yana-Kolyma Lowland, Siberia	70.8°N	136.3°E	Peat	-486.9	GIN-5389	(54)
Yana-Kolyma Lowland, Siberia	70.8°N	136.3°E	Peat	-527.9	GIN-5390b	(54)
Yana-Kolyma Lowland, Siberia	70.8°N	136.3°E	Peat	-582.7	GIN-5391	(54, 55)
Taymyr Peninsula, Siberia	72.4°N	99.7°E	Peat	-626.5	LZ-P5	(56)
Taymyr Peninsula, Siberia	72.4°N	99.7°E	Peat	-666.1	LZ-P6	(55, 56)
West Siberian Lowland	66.7°N	79.7°E	Peat	-437.1	CAMS-24132	(57)
West Siberian Lowland	66.7°N	79.7°E	Peat	-461.1	CAMS-24133	(57)
West Siberian Lowland	66.7°N	79.7°E	Peat	-575.2	CAMS-24134	(57)
West Siberian Lowland	66.7°N	79.7°E	Peat	-649.3	CAMS-2427	(57)
West Siberian Lowland	66.7°N	79.7°E	Peat	-683.7	CAMS-2428	(55, 57)
Yamal Peninsula, Siberia	70.0°N	72.0°E	Peat	-626.0	Hel-3945	(58)
Yamal Peninsula, Siberia	70.0°N	72.0°E	Peat	-631.5	Hel-4061	(58)
Yamal Peninsula, Siberia	70.0°N	72.0°E	Peat	-621.7	Hel-4062	(58)
Yamal Peninsula, Siberia	70.0°N	72.0°E	Peat	-642.8	Hel-3946	(58)
Yamal Peninsula, Siberia	70.0°N	72.0°E	Peat	-641.0	Hel-3947	(58)
Yamal Peninsula, Siberia	70.0°N	72.0°E	Peat	-643.2	Hel-4035	(58)
Yamal Peninsula, Siberia	70.0°N	72.0°E	Peat	-630.1	Hel-4047	(58)
Yamal Peninsula, Siberia	70.0°N	72.0°E	Peat	-641.0	Hel-4063	(58)
Yamal Peninsula, Siberia	70.0°N	72.0°E	Peat	-637.9	Hel-4036	(58)
Yamal Peninsula, Siberia	70.0°N	72.0°E	Peat	-638.3	Hel-4024	(58)
Yamal Peninsula, Siberia	70.0°N	72.0°E	Peat	-642.4	Hel-4037	(58)
Yamal Peninsula, Siberia	70.0°N	72.0°E	Peat	-645.5	Hel-4064	(58)
Yamal Peninsula, Siberia	70.0°N	72.0°E	Peat	-652.4	Hel-4025	(58)
Yamal Peninsula, Siberia	70.0°N	72.0°E	Peat	-653.7	Hel-4060	(58)
Yamal Peninsula, Siberia	70.0°N	72.0°E	Peat	-668.5	Hel-4038	(58)
Yamal Peninsula, Siberia	70.0°N	72.0°E	Peat	-650.3	Hel-4065	(58)
Yamal Peninsula, Siberia	70.0°N	72.0°E	Peat	-644.6	Hel-4039	(58)
Yamal Peninsula, Siberia	70.0°N	72.0°E	Peat	-642.4	Hel-4048	(58)
Yamal Peninsula, Siberia	70.0°N	72.0°E	Peat	-655.9	Hel-4040	(58)
Yamal Peninsula, Siberia	70.0°N	72.0°E	Peat	-649.4	Hel-4026	(58)
Yamal Peninsula, Siberia	70.0°N	72.0°E	Peat	-643.7	Hel-4049	(58)
Yamal Peninsula, Siberia	70.0°N	72.0°E	Peat	-647.7	Hel-4027	(58)
Yamal Peninsula, Siberia	70.0°N	72.0°E	Peat	-641.0	Hel-4028	(58)
Yamal Peninsula, Siberia	70.0°N	72.0°E	Peat	-638.8	Hel-4050	(58)
Yamal Peninsula, Siberia	70.0°N	72.0°E	Peat	-647.2	Hel-4029	(58)
Yamal Peninsula, Siberia	70.0°N	72.0°E	Peat	-645.9	Hel-4066	(58)
Yamal Peninsula, Siberia	70.0°N	72.0°E	Peat	-659.7	Hel-4041	(58)
Yamal Peninsula, Siberia	70.0°N	72.0°E	Peat	-654.6	Hel-3948	(58)
Yamal Peninsula, Siberia	70.0°N	72.0°E	Peat	-648.5	Hel-4051	(58)
Yamal Peninsula, Siberia	70.0°N	72.0°E	Peat	-650.7	Hel-4042	(58)
Yamal Peninsula, Siberia	70.0°N	72.0°E	Peat	-644.6	Hel-4030	(58)
Yamal Peninsula, Siberia	70.0°N	72.0°E	Peat	-647.2	Hel-4067	(58)
Yamal Peninsula, Siberia	70.0°N	72.0°E	Peat	-659.3	Hel-3949	(58)

Yamal Peninsula, Siberia	70.0°N	72.0°E	Peat	-665.2	Hel-3944	(58)
Yamal Peninsula, Siberia	70.0°N	72.0°E	Peat	-667.3	Hel-4034	(55, 58)
Yamal Peninsula, Siberia	67.0°N	69.0°E	Peat	-535.5	Hel-4138	(58)
Yamal Peninsula, Siberia	67.0°N	69.0°E	Peat	-546.4	Hel-4139	(58)
Yamal Peninsula, Siberia	67.0°N	69.0°E	Peat	-554.7	Hel-4137	(58)
Yamal Peninsula, Siberia	67.0°N	69.0°E	Peat	-561.3	Hel-4136	(58)
Yamal Peninsula, Siberia	67.0°N	69.0°E	Peat	-585.2	Hel-4135	(58)
Yamal Peninsula, Siberia	67.0°N	69.0°E	Peat	-582.1	Hel-4133	(58)
Yamal Peninsula, Siberia	67.0°N	69.0°E	Peat	-591.4	Hel-4134	(58)
Yamal Peninsula, Siberia	67.0°N	69.0°E	Peat	-588.8	Hel-4141	(58)
Yamal Peninsula, Siberia	67.0°N	69.0°E	Peat	-587.8	Hel-4142	(58)
Yamal Peninsula, Siberia	67.0°N	69.0°E	Peat	-605.4	Hel-4140	(55, 58)
West Siberian Lowland	66.5°N	67.0°E	Peat	-254.5	Hel-4342	(58)
West Siberian Lowland	66.5°N	67.0°E	Peat	-296.0	Hel-4343	(58)
West Siberian Lowland	66.5°N	67.0°E	Peat	-401.2	Hel-4344	(58)
West Siberian Lowland	66.5°N	67.0°E	Peat	-455.9	Hel-4340	(55, 58)
West Siberian Lowland	66.5°N	67.0°E	Peat	-461.3	Hel-4345	(58)
West Siberian Lowland	66.5°N	67.0°E	Peat	-444.3	Hel-4341	(58)
West Siberian Lowland	66.5°N	67.0°E	Peat	-426.0	Hel-4346	(58)
West Siberian Lowland	66.5°N	67.0°E	Peat	-451.9	Hel-4338	(58)
West Siberian Lowland	66.5°N	67.0°E	Peat	-445.7	Hel-4339	(55, 58)
Plaxhanski Yar, eastern Siberia	68.7°N	160.2°E	THK	-145.4	OS-96395	(59)
Cape Chukochi, eastern Siberia	70.0°N	160.0°E	THK	-233.1	UCIAMS-70668	(59)
Cherskiy, eastern Siberia	68.8°N	161.3°E	THK	-275.8	OS-96422	(59)
Cherskiy, eastern Siberia	68.8°N	161.3°E	THK	-307.1	UCIAMS-7058	(59) Min. age
Cape Chukochi, eastern Siberia	70.0°N	160.0°E	THK	-314.8	UCIAMS-70683	(59)
Cherskiy, eastern Siberia	68.8°N	161.3°E	THK	-322.9	UCIAMS-7069	(59) Min. age
Duvanny Yar, eastern Siberia	68.6°N	159.2°E	THK	-331.3	OS-96396	(59) Min. age
Duvanny Yar, eastern Siberia	68.6°N	159.2°E	THK	-450.0	OS-96397	(59) Min. age
Duvanny Yar, eastern Siberia	68.6°N	159.2°E	THK	-488.9	OS-96426	(59)
Duvanny Yar, eastern Siberia	68.6°N	159.2°E	THK	-489.6	OS-96412	(59)
Duvanny Yar, eastern Siberia	68.6°N	159.2°E	THK	-528.7	OS-96389	(59) Min. age
Duvanny Yar, eastern Siberia	68.6°N	159.2°E	THK	-630.2	OS-96749	(59)
Plaxhanski Yar, eastern Siberia	68.7°N	160.2°E	THK	-677.2	OS-96429	(59) Min. age
Duvanny Yar, eastern Siberia	68.6°N	159.2°E	THK	-671.9	OS-96469	(59) Min. age
Plaxhanski Yar, eastern Siberia	68.7°N	160.2°E	THK	-687.4	OS-96728	(59)
Duvanny Yar, eastern Siberia	68.6°N	159.2°E	THK	-756.9	OS-96398	(59)
Duvanny Yar, eastern Siberia	68.6°N	159.2°E	THK	-798.3	OS-96729	(59)
Anuiy	68.3°N	161.5°E	THK	-816.3	OS-96392	(59)
Bolshoy Lyakhovsky Island	73.3°N	141.5°E	THK	-373.6	KIA 12445	(60)
Bolshoy Lyakhovsky Island	73.3°N	141.5°E	THK	-634.1	KIA 12544	(59, 60)
Bolshoy Lyakhovsky Island	73.3°N	141.5°E	THK	-374.0	KI-4849	(60)
Bolshoy Lyakhovsky Island	73.3°N	141.5°E	THK	-733.7	KI-4850	(60)
Bolshoy Lyakhovsky Island	73.3°N	141.5°E	THK	-631.4	KIA 11476	(60)
Bolshoy Lyakhovsky Island	73.3°N	141.5°E	THK	-651.5	KIA 11448	(60)
Bolshoy Lyakhovsky Island	73.3°N	141.5°E	THK	-640.9	KI-4847	(60)
Bolshoy Lyakhovsky Island	73.3°N	141.5°E	THK	-647.1	KIA 12552	(60)
Bolshoy Lyakhovsky Island	73.3°N	141.5°E	THK	-688.8	KIA 12551	(59, 60)
Bolshoy Lyakhovsky Island	73.3°N	141.5°E	THK	-651.0	KI-4853	(60)
Bolshoy Lyakhovsky Island	73.3°N	141.5°E	THK	-673.7	KIA 11477	(60)
Bolshoy Lyakhovsky Island	73.3°N	141.5°E	THK	-666.8	KIA 12547	(60)
Bolshoy Lyakhovsky Island	73.3°N	141.5°E	THK	-731.3	KIA 25704	(60)
Bolshoy Lyakhovsky Island	73.3°N	141.5°E	THK	-757.5	KIA 11451	(60)
Bolshoy Lyakhovsky Island	73.3°N	141.5°E	THK	-764.8	KI-4851	(59, 60)
Oyogos Yar, eastern Siberia	72.7°N	143.6°E	THK	-632.2	KIA 25724	(61)
Oyogos Yar, eastern Siberia	72.7°N	143.6°E	THK	-648.4	KIA 25725	(61)

Oyogos Yar, eastern Siberia	72.7°N	143.6°E	THK	-700.9	KIA 25726	(61)
Cape Mamontov Klyk	73.6°N	117.2°E	THK	-375.1	KIA 25110	(62)
Cape Mamontov Klyk	73.6°N	117.2°E	THK	-174.1	KIA 29835	(62)
Cape Mamontov Klyk	73.6°N	117.2°E	THK	-587.7	KIA 29834	(62)
Lake El'gene Kyuele	71.3°N	125.6°E	THK	-115.7	Poz-50561	(63)
Lake El'gene Kyuele	71.3°N	125.6°E	THK	-128.8	Poz-50563	(63)
Lake El'gene Kyuele	71.3°N	125.6°E	THK	-603.8	Poz-49475	(63)
Lake El'gene Kyuele	71.3°N	125.6°E	THK	-624.0	Poz-49477	(63)
Lake El'gene Kyuele	71.3°N	125.6°E	THK	-742.5	Poz-49478	(63)
Kolyma Lowland			THK	-319.8	MAG-476	(64)
Kolyma Lowland			THK	-592.7	MAG-475	(64)
Kolyma Lowland			THK	-611.0	MAG-474	(64)
Kolyma Lowland			THK	-632.7	MAG-473	(64)
Kolyma Lowland			THK	-649.7	MAG-479	(64)
Kolyma Lowland			THK	-653.1	MAG-472	(64)
Buor-Khaya Peninsula	71.6°N	132.2°E	THK	-451.1	Poz-42080	(14)
Buor-Khaya Peninsula	71.6°N	132.2°E	THK	-370.9	Poz-42072	(14)
Buor-Khaya Peninsula	71.6°N	132.2°E	THK	-639.6	Poz-42073	(14)
Bolshoy Lyakhovsky Island	73.3°N	141.5°E	THK	-393.5	KIA 36692	(65)
Bolshoy Lyakhovsky Island	73.3°N	141.5°E	THK	-610.9	KIA 35227	(65)
Bolshoy Lyakhovsky Island	73.3°N	141.5°E	THK	-766.0	KIA 35226	(65)
Bolshoy Lyakhovsky Island	73.3°N	141.5°E	THK	-717.3	KIA 36691	(65)
Bolshoy Lyakhovsky Island	73.3°N	141.5°E	THK	-684.9	KIA 35225	(65)
Bolshoy Lyakhovsky Island	73.3°N	141.5°E	THK	-589.5	KIA 36690	(65)
Bolshoy Lyakhovsky Island	73.3°N	141.5°E	THK	-754.1	KIA 35224	(65)
Bolshoy Lyakhovsky Island	73.3°N	141.5°E	THK	-773.2	KIA 35223	(65)
Oyogos Yar, eastern Siberia	72.7°N	143.6°E	THK	-343.6	KIA 35234	(65)
Oyogos Yar, eastern Siberia	72.7°N	143.6°E	THK	-648.2	KIA 35233	(65)
Oyogos Yar, eastern Siberia	72.7°N	143.6°E	THK	-644.9	KIA 35232	(65)
Oyogos Yar, eastern Siberia	72.7°N	143.6°E	THK	-713.5	KIA 36687	(65)
Oyogos Yar, eastern Siberia	72.7°N	143.6°E	THK	-752.1	KIA 36686	(65)
Oyogos Yar, eastern Siberia	72.7°N	143.6°E	THK	-738.6	KIA 36688	(65)
Oyogos Yar, eastern Siberia	72.7°N	143.6°E	THK	-776.9	KIA 35230	(65)
Mean (peat)				-565.8		
Standard deviation (peat)				125.1		
Number of observations (peat)				70		
Mean (THK)				-569.2		
Standard deviation (THK)				184.7		
Number of observations (THK)				68		
Mean (all)				-567.5		
Standard deviation (all)				156.7		
Number of observations (all)				138		

Table S5. Database of $\Delta^{14}C$ values of Ice Complex Deposit exposures.

Table S5. Database of $\Delta^{14}C$ values of Ice Complex Deposit exposures in Siberia. Lat., latitude; Long., longitude.

Location	Lat.	Long.	$\Delta^{14}C$ (‰)	^{14}C Lab ID	Reference
Bykovsky Peninsula, Siberia	71.8°N	129.3°E	-997.3	KIA 6737	(66)
Bykovsky Peninsula, Siberia	71.8°N	129.3°E	-992.7	KI-4427.023	(66) Min. age
Bykovsky Peninsula, Siberia	71.8°N	129.3°E	-989.2	KI-4447.02	(66)
Bykovsky Peninsula, Siberia	71.8°N	129.3°E	-987.3	KIA 8164	(66)
Bykovsky Peninsula, Siberia	71.8°N	129.3°E	-841.1	KIA 10355	(66)
Batagay, Siberia	67.6°N	134.8°E	-984.6	Poz-79751, Poz-80390	(67)
Batagay, Siberia	67.6°N	134.8°E	-961.9	Poz-77152	(67)
Batagay, Siberia	67.6°N	134.8°E	-794.8	Poz-79756	(67)
Batagay, Siberia	67.6°N	134.8°E	-997.5	Poz-79753	(67)
Batagay, Siberia	67.6°N	134.8°E	-998.3	Poz-79754	(67)
Batagay, Siberia	67.6°N	134.8°E	-997.8	Poz-79755	(67)
Eastern Siberia	69.7°N	162.5°E	-993.0		(43)
Eastern Siberia	68.8°N	161.4°E	-964.0		(43)
Lena Delta, Siberia	72.5°N	128.0°E	-833.1	GIN-4115	(68)
Bykovsky Peninsula, Siberia	71.8°N	129.3°E	-764.0	KIA 25714	(69)
Bykovsky Peninsula, Siberia	71.8°N	129.3°E	-768.8	KIA 20692	(69)
Bykovsky Peninsula, Siberia	71.8°N	129.3°E	-781.0	KIA 20693	(69)
Bykovsky Peninsula, Siberia	71.8°N	129.3°E	-729.2	KIA 25713	(69)
Bykovsky Peninsula, Siberia	71.8°N	129.3°E	-737.5	KIA 20698	(69)
Bykovsky Peninsula, Siberia	71.8°N	129.3°E	-755.5	KIA 20697	(69)
Bykovsky Peninsula, Siberia	71.8°N	129.3°E	-759.7	KIA 20696	(69)
Bykovsky Peninsula, Siberia	71.8°N	129.3°E	-718.2	KIA 20695	(69)
Bykovsky Peninsula, Siberia	71.8°N	129.3°E	-721.4	KIA 25712	(69)
Bykovsky Peninsula, Siberia	71.8°N	129.3°E	-720.7	KIA 20694	(69)
Bykovsky Peninsula, Siberia	71.8°N	129.3°E	-735.2	KIA 25715	(69)
Bykovsky Peninsula, Siberia	71.8°N	129.3°E	-800.4	KIA 25716	(69)
Bykovsky Peninsula, Siberia	71.8°N	129.3°E	-841.9	KIA 25717	(69)
Bykovsky Peninsula, Siberia	71.8°N	129.3°E	-910.5	KIA 8166	(69)
Bykovsky Peninsula, Siberia	71.8°N	129.3°E	-998.6	KIA 8165	(69)
Duvanny Yar, Siberia	68.6°N	159.2°E	-969.9	GIN-7697	(70)
Duvanny Yar, Siberia	68.6°N	159.2°E	-979.3	GIN-8016	(70)
Duvanny Yar, Siberia	68.6°N	159.2°E	-994.9	GIN-9596	(70)
Duvanny Yar, Siberia	68.6°N	159.2°E	-995.7	GIN-9595	(70)
Duvanny Yar, Siberia	68.6°N	159.2°E	-979.2	GIN-2280	(70)
Duvanny Yar, Siberia	68.6°N	159.2°E	-985.2	GIN-2279	(70)
Duvanny Yar, Siberia	68.6°N	159.2°E	-991.2	GIN-2277	(70)
Duvanny Yar, Siberia	68.6°N	159.2°E	-990.8	MSU-468	(70)
Duvanny Yar, Siberia	68.6°N	159.2°E	-991.2	GIN-1688	(70)
Duvanny Yar, Siberia	68.6°N	159.2°E	-989.9	MSU-469	(70)
Duvanny Yar, Siberia	68.6°N	159.2°E	-996.3	MSU-573	(70) Min. age
Cape Mamontov Klyk, Siberia	73.6°N	117.2°E	-996.1		(71) Min. age
Cape Mamontov Klyk, Siberia	73.6°N	117.2°E	-993.5		(71)
Cape Mamontov Klyk, Siberia	73.6°N	117.2°E	-990.2		(71) Min. age
Cape Mamontov Klyk, Siberia	73.6°N	117.2°E	-994.8		(71)
Cape Mamontov Klyk, Siberia	73.6°N	117.2°E	-996.0		(71)
Cape Mamontov Klyk, Siberia	73.6°N	117.2°E	-995.6		(71)
Cape Mamontov Klyk, Siberia	73.6°N	117.2°E	-995.6		(71)
Cape Mamontov Klyk, Siberia	73.6°N	117.2°E	-979.7		(71)
Cape Mamontov Klyk, Siberia	73.6°N	117.2°E	-953.6		(71)
Cape Mamontov Klyk, Siberia	73.6°N	117.2°E	-924.0		(71)

Cape Mamontov Klyk, Siberia	73.6°N	117.2°E	-872.9		(71)
Cape Mamontov Klyk, Siberia	73.6°N	117.2°E	-749.4		(71)
Cape Mamontov Klyk, Siberia	73.6°N	117.2°E	-966.5		(71)
Cape Mamontov Klyk, Siberia	73.6°N	117.2°E	-950.9		(71)
Cape Mamontov Klyk, Siberia	73.6°N	117.2°E	-934.9		(71)
Cape Mamontov Klyk, Siberia	73.6°N	117.2°E	-919.5		(71)
Cape Mamontov Klyk, Siberia	73.6°N	117.2°E	-905.8		(71)
Cape Mamontov Klyk, Siberia	73.6°N	117.2°E	-890.4		(71)
Cape Mamontov Klyk, Siberia	73.6°N	117.2°E	-870.3		(71)
Cape Mamontov Klyk, Siberia	73.6°N	117.2°E	-837.6		(71)
Duvanny Yar, Siberia	68.6°N	159.2°E	-878.1	Poz-32563	(70)
Duvanny Yar, Siberia	68.6°N	159.2°E	-891.7	Poz-32564	(70)
Duvanny Yar, Siberia	68.6°N	159.2°E	-915.4	Poz-32457	(70)
Duvanny Yar, Siberia	68.6°N	159.2°E	-921.1	Poz-32458	(70)
Duvanny Yar, Siberia	68.6°N	159.2°E	-924.2	Poz-32490	(70)
Duvanny Yar, Siberia	68.6°N	159.2°E	-930.8	Poz-32554	(70)
Duvanny Yar, Siberia	68.6°N	159.2°E	-932.5	Poz-32555	(70)
Duvanny Yar, Siberia	68.6°N	159.2°E	-942.6	Poz-32557	(70)
Duvanny Yar, Siberia	68.6°N	159.2°E	-946.9	Poz-32558	(70)
Duvanny Yar, Siberia	68.6°N	159.2°E	-947.6	Poz-32559	(70)
Duvanny Yar, Siberia	68.6°N	159.2°E	-952.6	Poz-32560	(70)
Duvanny Yar, Siberia	68.6°N	159.2°E	-956.0	Poz-32561	(70)
Duvanny Yar, Siberia	68.6°N	159.2°E	-957.6	Poz-32562	(70)
Duvanny Yar, Siberia	68.6°N	159.2°E	-978.3	Poz-32417	(70)
Duvanny Yar, Siberia	68.6°N	159.2°E	-981.7	Poz-32418	(70)
Duvanny Yar, Siberia	68.6°N	159.2°E	-980.8	Poz-32419	(70)
Duvanny Yar, Siberia	68.6°N	159.2°E	-980.1	Poz-32454	(70)
Duvanny Yar, Siberia	68.6°N	159.2°E	-979.6	Poz-32455	(70)
Duvanny Yar, Siberia	68.6°N	159.2°E	-985.6	Poz-32456	(70)
Duvanny Yar, Siberia	68.6°N	159.2°E	-981.7	Poz-32487	(70)
Duvanny Yar, Siberia	68.6°N	159.2°E	-983.9	Poz-32489	(70)
Duvanny Yar, Siberia	68.6°N	159.2°E	-985.8	Poz-32415	(70)
Duvanny Yar, Siberia	68.6°N	159.2°E	-985.0	Poz-32416	(70)
Duvanny Yar, Siberia	68.6°N	159.2°E	-994.7	Poz-32483	(70)
Duvanny Yar, Siberia	68.6°N	159.2°E	-994.9	Poz-32484	(70)
Duvanny Yar, Siberia	68.6°N	159.2°E	-996.0	Poz-32485	(70)
Duvanny Yar, Siberia	68.6°N	159.2°E	-996.0	Poz-32486	(70)
Duvanny Yar, Siberia	68.6°N	159.2°E	-996.0	Poz-32376	(70)
Duvanny Yar, Siberia	68.6°N	159.2°E	-997.6	Poz-32377	(70)
Duvanny Yar, Siberia	68.6°N	159.2°E	-996.6	Poz-32378	(70)
Duvanny Yar, Siberia	68.6°N	159.2°E	-996.8	Poz-32302	(70) Min. age
Duvanny Yar, Siberia	68.6°N	159.2°E	-997.3	Poz-32369	(70)
Duvanny Yar, Siberia	68.6°N	159.2°E	-996.3	Poz-32370	(70) Min. age
Duvanny Yar, Siberia	68.6°N	159.2°E	-997.6	Poz-32371	(70)
Duvanny Yar, Siberia	68.6°N	159.2°E	-997.5	Poz-32373	(70) Min. age
Duvanny Yar, Siberia	68.6°N	159.2°E	-996.3	Poz-32374	(70) Min. age
Duvanny Yar, Siberia	68.6°N	159.2°E	-996.3	Poz-32375	(70) Min. age
Duvanny Yar, Siberia	68.6°N	159.2°E	-997.5	Poz-32301	(70) Min. age
Duvanny Yar, Siberia	68.6°N	159.2°E	-997.9	Poz-32232	(70)
Duvanny Yar, Siberia	68.6°N	159.2°E	-996.3	Poz-32368	(70)
Duvanny Yar, Siberia	68.6°N	159.2°E	-997.8	Poz-32626	(70) Min. age
Duvanny Yar, Siberia	68.6°N	159.2°E	-996.3	Poz-32627	(70) Min. age
Duvanny Yar, Siberia	68.6°N	159.2°E	-997.5	Poz-32628	(70) Min. age
Duvanny Yar, Siberia	68.6°N	159.2°E	-997.7	Poz-32629	(70)
Duvanny Yar, Siberia	68.6°N	159.2°E	-980.6	Poz-32631	(70)
Duvanny Yar, Siberia	68.6°N	159.2°E	-983.3	Poz-32630	(70)

Duvanny Yar, Siberia	68.6°N	159.2°E	-984.7	Poz-32300	(70)
Duvanny Yar, Siberia	68.6°N	159.2°E	-917.7	GIN-7696	(70)
Oyogos Yar, Siberia	72.7°N	143.6°E	-989.7	KIA 25731	(61)
Eastern Siberia	62.5°N	135.6°E	-964.5		(72)
Eastern Siberia	62.5°N	135.6°E	-993.6		(72)
Eastern Siberia	62.5°N	135.6°E	-995.8		(72)
Eastern Siberia	62.5°N	135.6°E	-985.6		(72)
Eastern Siberia	62.5°N	135.6°E	-994.8	SI-1965	(72)
Eastern Siberia	62.5°N	135.6°E	-997.0	SI-1972	(72)
Eastern Siberia	62.5°N	135.6°E	-999.1		(72) Min. age
Eastern Siberia	62.5°N	135.6°E	-999.1		(72) Min. age
Eastern Siberia	62.5°N	135.6°E	-999.1		(72) Min. age
Eastern Siberia	62.5°N	135.6°E	-996.8	SI-1967	(72) Min. age
Eastern Siberia	62.5°N	135.6°E	-999.1		(72) Min. age
Eastern Siberia	62.5°N	135.6°E	-995.6	SI-1966	(72) Min. age
Eastern Siberia	62.5°N	135.6°E	-999.1		(72) Min. age
Bykovsky Peninsula, Siberia	71.8°N	129.3°E	-824.3	KIA 9194	(73)
Bykovsky Peninsula, Siberia	71.8°N	129.3°E	-882.6	KIA 9195	(73)
Bykovsky Peninsula, Siberia	71.8°N	129.3°E	-885.4	KIA 10356	(73)
Bykovsky Peninsula, Siberia	71.8°N	129.3°E	-910.5	KIA 9196	(73)
Bykovsky Peninsula, Siberia	71.8°N	129.3°E	-923.5	KIA 9197	(73)
Bykovsky Peninsula, Siberia	71.8°N	129.3°E	-882.6	KIA 8361	(73)
Bykovsky Peninsula, Siberia	71.8°N	129.3°E	-900.5	KIA 12508	(73)
Bykovsky Peninsula, Siberia	71.8°N	129.3°E	-936.2	KIA 10357	(73)
Bykovsky Peninsula, Siberia	71.8°N	129.3°E	-948.3	KIA 8362	(73)
Bykovsky Peninsula, Siberia	71.8°N	129.3°E	-948.6	KIA 10358	(73)
Bykovsky Peninsula, Siberia	71.8°N	129.3°E	-952.8	KIA 10359	(73)
Bykovsky Peninsula, Siberia	71.8°N	129.3°E	-958.8	KIA 10360	(73)
Bykovsky Peninsula, Siberia	71.8°N	129.3°E	-952.7	KIA 6721	(73)
Bykovsky Peninsula, Siberia	71.8°N	129.3°E	-970.0	KIA 10361	(73)
Bykovsky Peninsula, Siberia	71.8°N	129.3°E	-984.8	KIA 6713	(73)
Bykovsky Peninsula, Siberia	71.8°N	129.3°E	-971.3	KIA 6716	(73)
Bykovsky Peninsula, Siberia	71.8°N	129.3°E	-984.6	KIA 6712	(73)
Bykovsky Peninsula, Siberia	71.8°N	129.3°E	-971.4	PI 2008	(73)
Bykovsky Peninsula, Siberia	71.8°N	129.3°E	-987.3	KIA 6714	(73)
Bykovsky Peninsula, Siberia	71.8°N	129.3°E	-988.8	KIA 6711	(73)
Bykovsky Peninsula, Siberia	71.8°N	129.3°E	-986.9	KIA 6715	(73)
Bykovsky Peninsula, Siberia	71.8°N	129.3°E	-989.8	KIA 6708	(73)
Bykovsky Peninsula, Siberia	71.8°N	129.3°E	-990.1	KIA 6710	(73)
Bykovsky Peninsula, Siberia	71.8°N	129.3°E	-991.0	KIA 6709	(73)
Bykovsky Peninsula, Siberia	71.8°N	129.3°E	-986.8	KIA 6725	(73)
Bykovsky Peninsula, Siberia	71.8°N	129.3°E	-988.6	KIA 6707	(73)
Bykovsky Peninsula, Siberia	71.8°N	129.3°E	-996.3	KI-4427.0-13	(73) Min. age
Bykovsky Peninsula, Siberia	71.8°N	129.3°E	-994.3	KI-4447.0-29	(73)
Bykovsky Peninsula, Siberia	71.8°N	129.3°E	-994.6	KI-4427.0-33	(73) Min. age
Bykovsky Peninsula, Siberia	71.8°N	129.3°E	-994.5	KIA 6726	(73)
Bykovsky Peninsula, Siberia	71.8°N	129.3°E	-992.6	KIA 6706	(73)
Bykovsky Peninsula, Siberia	71.8°N	129.3°E	-996.0	KIA 6705	(73)
Bykovsky Peninsula, Siberia	71.8°N	129.3°E	-996.1	KIA 6704	(73)
Bykovsky Peninsula, Siberia	71.8°N	129.3°E	-997.5	KIA 6703	(73)
Bykovsky Peninsula, Siberia	71.8°N	129.3°E	-997.4	KIA 6702	(73)
Bykovsky Peninsula, Siberia	71.8°N	129.3°E	-996.4	KIA 6727	(73)
Bykovsky Peninsula, Siberia	71.8°N	129.3°E	-996.5	KIA 8160	(73)
Bykovsky Peninsula, Siberia	71.8°N	129.3°E	-995.1	KIA 6701	(73)
Bykovsky Peninsula, Siberia	71.8°N	129.3°E	-998.6	KIA 6729	(73)
Bykovsky Peninsula, Siberia	71.8°N	129.3°E	-999.3	KIA 6730	(73)

Bykovsky Peninsula, Siberia	71.8°N	129.3°E	-998.6	KIA 6731	(73) Min. age
Bykovsky Peninsula, Siberia	71.8°N	129.3°E	-998.9	KIA 12509	(73)
Bykovsky Peninsula, Siberia	71.8°N	129.3°E	-999.2	KIA 12510	(73)
Lena Delta, Siberia	72.9°N	123.3°E	-997.3	KIA 9898	(74) Min. age
Lena Delta, Siberia	72.9°N	123.3°E	-995.3	KIA 9899	(74)
Lena Delta, Siberia	72.9°N	123.3°E	-999.2	KIA 6753	(74) Min. age
Lena Delta, Siberia	72.9°N	123.3°E	-996.6	KI 4938	(74) Min. age
Lena Delta, Siberia	72.3°N	126.3°E	-995.2	KIA 6755	(74)
Lena Delta, Siberia	72.3°N	126.3°E	-879.9	KIA 12595	(74)
Lena Delta, Siberia	72.3°N	126.3°E	-984.6	KIA 12594	(74)
Lena Delta, Siberia	72.3°N	126.3°E	-991.3	KIA 12593	(74)
Lena Delta, Siberia	72.3°N	126.3°E	-996.1	KIA 12592	(74)
Lena Delta, Siberia	72.3°N	126.3°E	-986.1	GIN 110883	(74)
Lena Delta, Siberia	72.3°N	126.3°E	-998.1	KIA 12591	(74)
Lena Delta, Siberia	73.0°N	124.2°E	-998.6	KIA 12526	(74) Min. age
Lena Delta, Siberia	73.0°N	124.2°E	-962.9	IM 832	(74)
Lena Delta, Siberia	73.0°N	124.2°E	-956.7	LU 4408	(74)
Cape Mamontov Klyk, Siberia	73.6°N	117.2°E	-979.7	KIA 23773	(62)
Cape Mamontov Klyk, Siberia	73.6°N	117.2°E	-953.6	KIA 25090	(62)
Cape Mamontov Klyk, Siberia	73.6°N	117.2°E	-924.0	KIA 25091	(62)
Cape Mamontov Klyk, Siberia	73.6°N	117.2°E	-912.4	KIA 25092	(62)
Cape Mamontov Klyk, Siberia	73.6°N	117.2°E	-901.5	KIA 25093	(62)
Cape Mamontov Klyk, Siberia	73.6°N	117.2°E	-872.8	KIA 25094	(62)
Cape Mamontov Klyk, Siberia	73.6°N	117.2°E	-862.6	KIA 29831	(62)
Cape Mamontov Klyk, Siberia	73.6°N	117.2°E	-966.5	KIA 25097	(62)
Cape Mamontov Klyk, Siberia	73.6°N	117.2°E	-950.9	KIA 25098	(62)
Cape Mamontov Klyk, Siberia	73.6°N	117.2°E	-934.9	KIA 25099	(62)
Cape Mamontov Klyk, Siberia	73.6°N	117.2°E	-919.5	KIA 25100	(62)
Cape Mamontov Klyk, Siberia	73.6°N	117.2°E	-905.8	KIA 25101	(62)
Cape Mamontov Klyk, Siberia	73.6°N	117.2°E	-890.3	KIA 25102	(62)
Cape Mamontov Klyk, Siberia	73.6°N	117.2°E	-870.3	KIA 25103	(62)
Cape Mamontov Klyk, Siberia	73.6°N	117.2°E	-837.6	KIA 25104	(62)
Cape Mamontov Klyk, Siberia	73.6°N	117.2°E	-749.4	KIA 26107	(62)
Lena Delta, Siberia	73.0°N	124.2°E	-995.6	KIA 31029	(19) Min. age
Lena Delta, Siberia	73.0°N	124.2°E	-997.8	KIA 31030	(19) Min. age
Lena Delta, Siberia	73.0°N	124.2°E	-998.5	KIA 31031	(19) Min. age
Lena Delta, Siberia	73.0°N	124.2°E	-998.1	KIA 31032	(19) Min. age
Lena Delta, Siberia	73.0°N	124.2°E	-975.6	KIA 31033	(19)
Lena Delta, Siberia	73.0°N	124.2°E	-969.8	KIA 31034	(19)
Lena Delta, Siberia	73.0°N	124.2°E	-918.7	KIA 31035	(19)
Buor Khaya Gulf, Siberia	71.6°N	129.9°E	-992.4	KIA 25718	(19)
Buor Khaya Gulf, Siberia	71.6°N	129.9°E	-997.1	KIA 25720	(19)
Buor Khaya Gulf, Siberia	71.6°N	129.9°E	-995.2	KIA 25719	(19)
Buor Khaya Gulf, Siberia	71.6°N	129.9°E	-991.9	KIA 25721	(19)
Buor Khaya Gulf, Siberia	71.6°N	129.9°E	-993.5	KIA 25722	(19)
Buor Khaya Gulf, Siberia	71.6°N	129.9°E	-913.0	KIA 25723	(19)
Cape Svyatoy Nos, Siberia	72.8°N	140.9°E	-997.4	KIA 12517	(19) Min. age
Cape Svyatoy Nos, Siberia	72.8°N	140.9°E	-996.5	KIA 12518	(19)
Cape Svyatoy Nos, Siberia	72.8°N	140.9°E	-998.1	KIA 12516	(19) Min. age
Cape Svyatoy Nos, Siberia	72.8°N	140.9°E	-988.9	KIA 12515	(19)
Oyogos Yar, Siberia	72.7°N	143.5°E	-994.3	KIA 37630	(19)
Oyogos Yar, Siberia	72.7°N	143.5°E	-995.8	KIA 37631	(19)
Oyogos Yar, Siberia	72.7°N	143.5°E	-996.3	KIA 37632	(19)
Oyogos Yar, Siberia	72.7°N	143.5°E	-997.6	KIA 37633	(19)
Oyogos Yar, Siberia	72.7°N	143.5°E	-993.9	KIA 37634	(19)
Oyogos Yar, Siberia	72.7°N	143.5°E	-996.3	KIA 37635	(19)

Oyogos Yar, Siberia	72.7°N	143.5°E	-991.9	KIA 37636	(19)
Oyogos Yar, Siberia	72.7°N	143.5°E	-986.7	KIA 37637	(19)
Oyogos Yar, Siberia	72.7°N	143.5°E	-982.0	KIA 37638	(19)
Bykovsky Peninsula, Siberia	71.8°N	129.3°E	-970.2	IM-767	(69)
Bykovsky Peninsula, Siberia	71.8°N	129.3°E	-947.9	IM-766	(69)
Bykovsky Peninsula, Siberia	71.8°N	129.3°E	-932.6	LU-1328	(69)
Bykovsky Peninsula, Siberia	71.8°N	129.3°E	-983.7	LU-1130	(69)
Bykovsky Peninsula, Siberia	71.8°N	129.3°E	-993.3	GIN-4597	(69)
Bykovsky Peninsula, Siberia	71.8°N	129.3°E	-993.5	GIN-4593	(69)
Bykovsky Peninsula, Siberia	71.8°N	129.3°E	-993.8	GIN-4391	(69)
Buor Khaya Peninsula, Siberia	71.6°N	132.2°E	-976.6	Poz-42074	(14)
Buor Khaya Peninsula, Siberia	71.6°N	132.2°E	-986.7	Poz-42075	(14)
Buor Khaya Peninsula, Siberia	71.6°N	132.2°E	-994.3	Poz-42076	(14)
Buor Khaya Peninsula, Siberia	71.6°N	132.2°E	-996.3	Poz-42077	(14)
Buor Khaya Peninsula, Siberia	71.6°N	132.2°E	-995.3	Poz-42078	(14)
Buor Khaya Peninsula, Siberia	71.6°N	132.2°E	-997.8	Poz-42081	(14)
Buor Khaya Peninsula, Siberia	71.6°N	132.2°E	-997.5	Poz-42082	(14) Min. age
Buor Khaya Peninsula, Siberia	71.6°N	132.2°E	-998.9	Poz-42083	(14) Min. age
Buor Khaya Peninsula, Siberia	71.6°N	132.2°E	-997.8	Poz-42084	(14) Min. age
Buor Khaya Peninsula, Siberia	71.6°N	132.2°E	-998.9	Poz-42085	(14) Min. age
Duvanny Yar, Siberia	68.6°N	159.2°E	-950.2	SOAN-2302	(70)
Duvanny Yar, Siberia	68.6°N	159.2°E	-961.2	Lu-1675	(70)
Duvanny Yar, Siberia	68.6°N	159.2°E	-985.6	SOAN-2303	(70)
Duvanny Yar, Siberia	68.6°N	159.2°E	-986.2	Lu-1674	(70)
Duvanny Yar, Siberia	68.6°N	159.2°E	-995.2	Lu-1676	(70)
Duvanny Yar, Siberia	68.6°N	159.2°E	-997.6	SOAN-2304	(70)
Duvanny Yar, Siberia	68.6°N	159.2°E	-998.0	SOAN-2305	(70) Min. age
Duvanny Yar, Siberia	68.6°N	159.2°E	-998.7	Lu-1678	(70) Min. age
Duvanny Yar, Siberia	68.6°N	159.2°E	-986.8	GIN-4434	(70)
Duvanny Yar, Siberia	68.6°N	159.2°E	-996.5	GIN-3860	(70)
Duvanny Yar, Siberia	68.6°N	159.2°E	-998.0	GIN-3866	(70) Min. age
Duvanny Yar, Siberia	68.6°N	159.2°E	-995.9	GIN-4003	(70)
Duvanny Yar, Siberia	68.6°N	159.2°E	-984.6	GIN-4006	(70)
Duvanny Yar, Siberia	68.6°N	159.2°E	-996.1	GIN-4000	(70)
Duvanny Yar, Siberia	68.6°N	159.2°E	-988.0	GIN-3999	(70)
Duvanny Yar, Siberia	68.6°N	159.2°E	-998.6	GIN-3857	(70) Min. age
Duvanny Yar, Siberia	68.6°N	159.2°E	-996.4	GIN-3852	(70)
Duvanny Yar, Siberia	68.6°N	159.2°E	-989.8	GIN-3997	(70)
Eastern Siberia	70.6°N	134.3°E	-993.2	GIN-4983	(75) Min. age
Eastern Siberia	70.6°N	134.3°E	-995.7	GIN-4978	(75) Min. age
Eastern Siberia	70.6°N	134.3°E	-994.0	GIN-4977	(75)
Eastern Siberia	70.6°N	134.3°E	-993.6	GIN-4964	(75)
Eastern Siberia	70.6°N	134.3°E	-988.3	GIN-4979	(75)
Eastern Siberia	70.6°N	134.3°E	-994.9	GIN-4982	(75)
Eastern Siberia	70.6°N	134.3°E	-984.3	GIN-4987	(75)
Eastern Siberia	70.6°N	134.3°E	-992.0	GIN-4965	(75)
Eastern Siberia	70.6°N	134.3°E	-990.9	GIN-4981	(75)
Gydansky Peninsula, Siberia	70.9°N	78.5°E	-784.9	GIN-3597	(75)
Gydansky Peninsula, Siberia	70.9°N	78.5°E	-822.7	GIN-3591	(75)
Gydansky Peninsula, Siberia	70.9°N	78.5°E	-834.4	GIN-3592	(75)
Gydansky Peninsula, Siberia	70.9°N	78.5°E	-817.1	GIN-3595	(75)
Gydansky Peninsula, Siberia	70.9°N	78.5°E	-838.3	GIN-3609	(75)
Gydansky Peninsula, Siberia	70.9°N	78.5°E	-842.7	GIN-3603	(75)
Gydansky Peninsula, Siberia	70.9°N	78.5°E	-779.3	GIN-3611	(75)
Gydansky Peninsula, Siberia	70.9°N	78.5°E	-805.3	GIN-3608	(75)
Gydansky Peninsula, Siberia	70.9°N	78.5°E	-862.5	GIN-3585	(75)

Duvanny Yar, Siberia	68.6°N	159.1°E	-995.1	GIN-3862	(75)
Duvanny Yar, Siberia	68.6°N	159.1°E	-991.2	GIN-3864	(75)
Duvanny Yar, Siberia	68.6°N	159.1°E	-987.4	GIN-3865	(75)
Duvanny Yar, Siberia	68.6°N	159.1°E	-991.1	GIN-4015	(75)
Duvanny Yar, Siberia	68.6°N	159.1°E	-984.4	GIN-4018	(75)
Duvanny Yar, Siberia	68.6°N	159.1°E	-985.2	GIN-3861	(75)
Duvanny Yar, Siberia	68.6°N	159.1°E	-968.0	GIN-4016	(75)
Duvanny Yar, Siberia	68.6°N	159.1°E	-935.7	GIN-4017	(75)
Duvanny Yar, Siberia	68.6°N	159.1°E	-912.0	GIN-3868	(75)
Duvanny Yar, Siberia	68.6°N	159.1°E	-804.8	EP-941555	(75)
Eastern Siberia	62.5°N	151.4°E	-981.7	GIN-8922	(76)
Eastern Siberia	62.5°N	151.4°E	-980.0	GIN-8925	(76)
Eastern Siberia	62.5°N	151.4°E	-994.7	GIN-8929	(76)
Eastern Siberia	62.5°N	151.4°E	-988.0	GIN-8930	(76)
Yamal Peninsula	70.0°N	72.0°E	-765.6	Hel-3942	(77)
Yamal Peninsula	70.0°N	72.0°E	-884.3	Hel-4023	(77)
Yamal Peninsula	70.0°N	72.0°E	-941.0	GIN-2473	(77)
Yamal Peninsula	70.0°N	72.0°E	-940.3	GIN-2475	(77)
Yamal Peninsula	70.0°N	72.0°E	-939.6	GIN-8931	(77)
Yamal Peninsula	70.0°N	72.0°E	-942.1	Hel-4046	(77)
Yamal Peninsula	70.0°N	72.0°E	-946.6	GIN-2474	(77)
Yamal Peninsula	70.0°N	72.0°E	-952.6	Hel-4043	(77)
Yamal Peninsula	70.0°N	72.0°E	-957.3	Hel-4056	(77)
Yamal Peninsula	70.0°N	72.0°E	-969.1	Hel-3943	(77)
Yamal Peninsula	70.0°N	72.0°E	-951.6	GIN-2476	(77)
Yamal Peninsula	70.0°N	72.0°E	-974.7	GIN-8936	(77)
Yamal Peninsula	70.0°N	72.0°E	-976.5	GIN-2477	(77)
Yamal Peninsula	70.0°N	72.0°E	-979.5	Hela-201	(77)
Yamal Peninsula	70.0°N	72.0°E	-989.8	Hel-3950	(77)
Duvanny Yar, Siberia	68.6°N	159.1°E	-892.3	MAG-592	(78)
Duvanny Yar, Siberia	68.6°N	159.1°E	-912.1	GIN-4016	(78)
Duvanny Yar, Siberia	68.6°N	159.1°E	-971.8	GIN-3867	(78)
Duvanny Yar, Siberia	68.6°N	159.1°E	-976.0	GIN-4588	(78)
Duvanny Yar, Siberia	68.6°N	159.1°E	-976.6	GIN-3998	(78)
Duvanny Yar, Siberia	68.6°N	159.1°E	-987.9	GIN-3996	(78)
Lena Delta, Siberia	72.3°N	126.3°E	-983.5	KIA 30236	(79)
Lena Delta, Siberia	72.3°N	126.3°E	-981.4	KIA 30237	(79)
Lena Delta, Siberia	72.3°N	126.3°E	-987.0	KIA 30238	(79)
Lena Delta, Siberia	72.3°N	126.3°E	-993.5	KIA 30240	(79)
Lena Delta, Siberia	72.3°N	126.3°E	-993.2	KIA 30239	(79)
Lena Delta, Siberia	72.3°N	126.3°E	-994.1	KIA 30241	(79)
Lena Delta, Siberia	72.3°N	126.3°E	-994.2	KIA 31052	(79)
Oyogos Yar, Siberia	72.7°N	143.6°E	-997.8	Poz-51638	(80) Min. age
Oyogos Yar, Siberia	72.7°N	143.6°E	-998.3	Poz-51637	(80) Min. age
Eastern Siberia	63.6°N	130.0°E	-804.4	KIA 19138	(81)
Eastern Siberia	63.6°N	130.0°E	-920.5	KIA 19138	(81)
Eastern Siberia	63.6°N	130.0°E	-771.5	Erl. 6152	(81)
Eastern Siberia	63.6°N	130.0°E	-890.5	Erl. 6154	(81)
Eastern Siberia	63.6°N	130.0°E	-910.2	Erl. 6157	(81)
Eastern Siberia	63.6°N	130.0°E	-975.6	KIA 19140	(81)
Eastern Siberia	63.6°N	130.0°E	-996.8	Erl. 5203	(81) Min. age
Eastern Siberia	63.6°N	130.0°E	-997.2	KIA 18805	(81)
Eastern Siberia	63.6°N	130.0°E	-998.0	KIA 18805	(81)
Eastern Siberia	63.6°N	130.0°E	-997.5	Erl. 5203	(81) Min. age
Eastern Siberia	63.6°N	130.0°E	-998.3	Erl. 5204	(81) Min. age
Mean			-954.8		

Standard deviation	65.8
Number of observations	329

Table S6. DOC and POC fluxes, $\delta^{13}C$ and $\Delta^{14}C$ values in Ob, Yenisey, Lena, and Kolyma.

Table S6. DOC and POC fluxes, $\delta^{13}C$ and $\Delta^{14}C$ values in Ob, Yenisey, Lena, and Kolyma at individual sampling dates between 2003 and 2013. Data are also available on the ARCTIC-GRO homepage (www.arcticgreatrivers.org).

River	Date	DOC flux kg s^{-1}	DOC $\delta^{13}C$ ‰	DOC $\Delta^{14}C$ ‰	POC flux kg s^{-1}	POC $\delta^{13}C$ ‰	POC $\Delta^{14}C$ ‰
Ob	16/07/2003	342.1	-25.0	24.9	38.4	-29.7	
Ob	05/04/2004	19.5	-26.7	-114.0	4.0	-38.0	-264.0
Ob	15/06/2004	300.2	-27.9	52.0	75.2	-29.8	-214.4
Ob	17/06/2004	300.2	-28.0	71.0	78.7	-31.5	-205.8
Ob	28/07/2004	312.0	-26.8	43.0	42.9	-31.1	-236.1
Ob	11/08/2004	160.6	-26.0	22.0	21.0	-32.7	-150.0
Ob	11/10/2004	76.7	-26.0	-35.0	7.7	-33.6	-206.9
Ob	14/10/2004	80.6	-27.7	-29.0	8.4	-33.7	-206.7
Ob	15/03/2005	31.0	-29.1	-27.0	3.1	-35.4	-270.7
Ob	04/06/2005	341.0	-27.9	43.0	57.1	-29.9	-267.9
Ob	06/06/2005	355.0	-27.3	67.0	42.8	-30.3	
Ob	28/06/2005	297.8	-28.0	53.0	47.3	-31.5	-225.8
Ob	14/07/2005	292.6	-26.5	46.0	50.5	-31.7	-240.6
Ob	05/09/2005	98.6	-27.7	8.0	11.9	-32.5	-234.8
Ob	17/09/2005	91.6	-27.5	-1.0	10.0	-32.6	-235.3
Ob	07/06/2006	326.6				-31.3	-175.5
Ob	23/11/2006	62.0				-33.4	
Ob	02/06/2009	294.0	-26.4	30.1	79.5	-30.1	-180.4
Ob	07/06/2009	306.9	-12.1	2.6	63.9	-30.0	-182.8
Ob	13/06/2009	272.6	-27.8	32.8	45.0	-29.9	-158.1
Ob	25/08/2009	141.8	-28.1	23.1	17.6	-30.8	-195.8
Ob	01/12/2009	37.6	-28.2	-46.1	2.7	-32.4	-161.4
Ob	31/05/2010	307.2	-28.1	34.3	79.7	-29.5	-286.7
Ob	04/06/2010	321.4	-28.4	21.2	100.5	-29.3	-164.1
Ob	09/06/2010	299.9	-25.0	23.6	82.6	-28.6	
Ob	13/08/2010	289.1	-24.9	12.2	35.3	-31.8	-300.5
Ob	12/12/2010	53.3	-28.9	-31.0	3.9	-31.5	-192.2
Ob	29/05/2011	378.8	-27.7	-21.5	59.1	-30.0	-188.0
Ob	05/06/2011	367.2	-27.8	45.0	37.3	-30.2	-193.7
Ob	10/06/2011	348.1		55.7	50.4	-30.4	-196.9
Ob	08/08/2011	159.6	-28.3	13.4	20.2	-30.9	-164.8
Ob	07/12/2011	55.0	-29.3	-45.5	2.5	-32.9	-210.5
Ob	06/06/2012	255.4	-27.1	33.8	32.8	-30.1	
Ob	22/08/2012	73.1	-25.8	-67.6	4.1	-29.5	
Ob	25/10/2012	67.0			9.1	-29.8	
Ob	08/12/2012	39.0	-28.9	-28.5	4.5	-25.9	
Ob	03/01/2013	29.4	-28.7	-62.0	4.7	-30.2	
Ob	24/03/2013	16.0	-29.9	-124.7	3.9	-34.8	
Ob	26/05/2013	199.2	-28.7	3.9	37.3	-29.8	
Ob	13/07/2013	434.9			32.4	-30.3	
Ob	25/09/2013	82.1			23.9	-29.7	
Ob	25/11/2013	50.2			12.9	-31.9	
Yenisey	25/07/2003				10.9	-31.6	
Yenisey	19/03/2004	21.4	-25.6	34.0	1.6	-28.4	-374.9
Yenisey	14/06/2004	1241.1	-25.5	107.0	80.8	-27.8	-148.5
Yenisey	16/06/2004	1171.8	-27.3	126.0	86.0	-29.4	-135.0
Yenisey	18/06/2004	1032.8	-27.5	125.0	73.4	-27.9	-133.2
Yenisey	25/08/2004	89.2	-26.7	50.0	4.4	-30.6	-277.2

Yenisey	01/10/2004	137.2	-26.1	51.0	5.8	-30.2	-306.6
Yenisey	02/10/2004	137.5	-26.5	51.0	5.6	-30.5	-283.0
Yenisey	26/03/2005	45.0	-21.7	-12.0	1.8	-30.7	-486.5
Yenisey	11/06/2005	753.5	-27.6	99.0	5.6	-30.1	-225.7
Yenisey	16/06/2005	518.1	-27.1	90.0	15.5	-29.9	-217.9
Yenisey	17/06/2005	463.8	-27.6	106.0	14.1	-29.9	-317.7
Yenisey	16/08/2005	81.5	-20.4	2.0	3.9	-33.4	-210.1
Yenisey	21/08/2005	87.7	-20.5	17.0	4.4	-33.0	-282.2
Yenisey	21/09/2005	81.1	-27.4	16.0	3.1	-31.8	-334.5
Yenisey	17/06/2006	1064.4				-27.6	
Yenisey	22/11/2006	94.9			2.3	-32.4	-394.6
Yenisey	17/06/2009	836.9	-27.5	79.8	46.1	-29.0	
Yenisey	21/06/2009	638.4	-27.4	98.6	45.7	-28.4	-234.1
Yenisey	27/06/2009	437.8	-27.9	103.8	24.6	-28.3	-296.9
Yenisey	08/08/2009	85.1	-27.6	50.8	5.6	-32.7	
Yenisey	30/11/2009	44.7			6.0	-27.0	
Yenisey	18/06/2010	695.5	-28.8	54.7	45.7	-27.6	-316.9
Yenisey	23/06/2010	546.0	-28.5	61.1	37.4	-28.8	-275.4
Yenisey	27/06/2010	435.5	-29.4	55.9	21.8	-29.6	-247.6
Yenisey	21/09/2010	207.1	-28.5	18.7	11.6	-29.9	-302.6
Yenisey	08/12/2010	48.4	-23.2	-46.6	1.6	-30.6	-373.2
Yenisey	17/06/2011	246.1	-19.0	-2.3	8.0	-32.0	-252.3
Yenisey	21/06/2011	196.2	-21.9	26.9	6.8	-32.8	-186.2
Yenisey	02/07/2011	133.7	-27.2	52.8	6.3	-33.1	-164.7
Yenisey	29/09/2011	88.2		29.1	3.1	-31.4	-120.0
Yenisey	02/12/2011	40.6	-27.1	23.9	1.8	-30.8	-239.3
Yenisey	20/06/2012	299.3			17.2	-28.7	
Yenisey	19/08/2012	41.8	-22.4	-53.8	6.6	-31.4	
Yenisey	19/10/2012	33.0		-28.2	3.7	-30.0	
Yenisey	29/12/2012	19.3	-28.8	-14.0	1.1	-28.3	
Yenisey	26/01/2013	19.9	-12.1	-112.9	1.4	-28.7	
Yenisey	24/03/2013	13.2	-27.4	-18.4	1.4	-27.5	
Yenisey	15/05/2013	102.7	-23.5	-3.8	9.2	-28.5	
Yenisey	19/07/2013	169.5			5.7	-31.5	
Yenisey	23/09/2013	106.5			5.8	-30.0	
Yenisey	30/11/2013	44.4			8.1	-28.3	
Lena	12/08/2003	116.8	-27.1		49.7	-25.2	
Lena	09/04/2004	15.5	-27.2	63.0	0.4	-31.5	-320.8
Lena	05/06/2004	749.4	-27.3	120.0	208.6	-27.7	-262.2
Lena	07/06/2004	960.7	-28.3	121.0	287.4	-27.4	-249.8
Lena	19/08/2004	237.9	-26.8	75.0	25.2	-29.6	-289.5
Lena	24/08/2004	202.2	-26.5	39.0	23.3	-30.0	-285.9
Lena	07/10/2004	163.7	-26.8	51.0	16.4	-29.9	-283.4
Lena	10/10/2004	193.8	-26.4	63.0	13.6	-30.3	-329.9
Lena	24/03/2005	32.3	-27.5	83.0	0.3	-35.2	-355.1
Lena	27/05/2005	464.3	-25.2	112.0	60.9	-27.8	-288.7
Lena	04/06/2005	1273.7	-27.3	87.0	117.7	-26.9	-257.7
Lena	06/08/2005	400.4	-27.1	87.0	44.6	-28.9	-336.4
Lena	14/08/2005	309.1	-27.1	73.0	34.7	-29.5	-324.2
Lena	09/10/2005	174.5	-27.0	62.0	21.5	-28.4	-355.7
Lena	10/10/2005	162.4	-27.5	61.0	18.8	-29.2	-353.7
Lena	06/06/2006	1215.6			191.6	-27.8	-398.2
Lena	14/11/2006	47.3			1.0	-36.0	
Lena	31/05/2009	763.9	-26.9	152.3	62.0	-27.1	-198.8
Lena	05/06/2009	1967.3			313.3	-27.2	-261.0
Lena	11/06/2009	1081.0	-27.1	101.6	153.4	-26.6	-270.9

Lena	22/08/2009	227.1	-27.2	58.9	35.1	-29.7	
Lena	18/11/2009	31.6	-27.2	58.8	0.8	-32.5	-185.8
Lena	29/05/2010	547.7	-27.2	81.9	53.4	-28.3	-229.9
Lena	04/06/2010	1851.5	-28.0	79.3	150.6	-29.1	-204.9
Lena	11/06/2010	888.4	-27.3	82.1	80.8	-27.0	-271.2
Lena	01/09/2010	172.8	-27.6	49.8	18.8	-28.0	-296.3
Lena	23/11/2010	49.7	-27.8	32.6	0.9	-32.6	-244.8
Lena	27/05/2011	2527.2	-25.8	83.5	191.2	-28.4	
Lena	02/06/2011	1291.5	-28.6	49.6	88.6	-29.4	-163.7
Lena	08/06/2011	763.8	-26.9	86.8	66.8	-28.9	
Lena	17/09/2011	165.6	-27.4	38.1	34.5	-29.5	-274.0
Lena	16/11/2011	42.7	-15.3	-49.8	0.9	-29.1	-167.4
Lena	06/06/2012	1394.9	-27.9	84.8			
Lena	20/08/2012				32.6	-30.5	
Lena	08/10/2012	154.4	-27.0	55.0	8.3	-29.2	
Lena	08/12/2012	20.0	-27.5	27.8	0.3	-30.3	
Lena	05/01/2013	42.1	-28.1	60.6	0.6	-32.3	
Lena	31/03/2013	12.1	-28.2	54.5	0.2	-34.0	
Lena	31/05/2013	2139.0			217.5	-26.9	
Lena	30/07/2013	459.2			59.7	-28.9	
Lena	20/09/2013	179.4			17.6	-27.1	
Lena	16/11/2013	31.4			0.6	-32.6	
Kolyma	26/08/2003	6.7	-27.5	-18.9	0.9	-33.0	
Kolyma	11/06/2004	198.1	-27.3	86.8	19.9	-26.5	-369.6
Kolyma	15/06/2004	147.7	-30.0	-3.9	17.6	-28.1	
Kolyma	25/06/2004	80.5	-27.4	44.8	8.9	-29.7	-375.1
Kolyma	15/07/2004	47.6	-27.4	55.4	4.1	-29.9	-410.6
Kolyma	10/08/2004	23.4	-26.7	-5.1	2.7	-29.7	-413.2
Kolyma	25/08/2004	31.7	-27.0	19.1	4.4	-28.8	-416.6
Kolyma	23/09/2004	27.1	-27.3	8.5	4.6	-29.5	-436.6
Kolyma	22/04/2005	1.1	-27.2	20.4	0.2	-34.6	-635.3
Kolyma	30/06/2005	42.9	-26.1	-12.7	4.7	-30.5	-396.7
Kolyma	19/07/2005	21.5	-26.9	12.3	7.1	-27.4	-422.8
Kolyma	14/08/2005	40.2	-27.3	49.9	8.3	-27.3	-574.3
Kolyma	27/08/2005	70.0	-27.5	73.5		-28.5	-526.1
Kolyma	12/09/2005	32.1	-27.3	40.5	3.3	-28.4	-497.1
Kolyma	29/09/2005	19.2	-24.2	-14.6	1.3	-29.9	-492.6
Kolyma	24/07/2006	42.3			1.8	-31.8	-390.7
Kolyma	20/11/2006	8.1			0.1	-35.1	-167.9
Kolyma	05/06/2009	137.0	-27.6	63.2	15.0	-27.4	-364.8
Kolyma	12/06/2009	101.0	-27.7	72.8	11.2	-27.3	-354.7
Kolyma	21/06/2009	45.5	-27.6	37.5	6.0	-28.0	-355.2
Kolyma	09/09/2009	32.7			7.0	-27.4	-422.2
Kolyma	08/11/2009	4.8	-26.8	-21.6	0.4	-35.5	-195.5
Kolyma	30/05/2010	325.7	-28.0	93.9	26.9	-27.6	-400.5
Kolyma	07/06/2010	140.4	-27.9	61.5	17.8	-28.0	-452.9
Kolyma	13/06/2010	78.8	-27.7	59.0	7.7	-29.5	-477.4
Kolyma	20/09/2010	26.3	-28.1	17.7	9.9	-28.7	-516.8
Kolyma	17/11/2010	4.4	-27.6	21.6	0.2	-30.6	-428.5
Kolyma	10/06/2011	321.9	-27.8	79.1	75.6	-28.3	-416.9
Kolyma	17/06/2011	219.4	-27.9	69.2	49.6	-28.0	-424.7
Kolyma	24/06/2011	179.8	-27.2	50.1	64.8	-28.5	-460.4
Kolyma	05/09/2011	26.9	-26.6	-28.3	6.1	-29.5	-495.5
Kolyma	05/11/2011	6.0		5.3	0.3	-33.4	-203.7
Kolyma	24/06/2012	23.0	-29.8	59.2	2.7	-31.9	
Kolyma	22/08/2012	24.0	-30.1	30.4	5.7	-28.5	

Kolyma	07/10/2012	7.5			0.6	-30.9
Kolyma	17/12/2012	0.8	-25.8	-4.3	0.0	-36.3
Kolyma	08/03/2013	0.4	-27.9	-5.0	0.0	-27.1
Kolyma	03/05/2013	0.4	-28.1	5.9	0.0	-30.3
Kolyma	27/07/2013	27.1			3.8	-28.8
Kolyma	20/09/2013	41.0			4.3	-27.4
Kolyma	13/11/2013	3.7			0.1	-30.7

Table S7. Statistical analysis of DOC and POC $\delta^{13}C$ and $\Delta^{14}C$ values.

Table S7. Statistical analysis of DOC and POC $\delta^{13}C$ and $\Delta^{14}C$ values. Flux-weighted differences between rivers and seasons were analyzed with two-way ANOVA followed by Tukey's HSD as post hoc test for individual rivers or seasons in R 3.5.1 (23) with the packages 'Hmisc' (24) and 'HH' (82). Differences were considered significant at $p < 0.05$ (n.s., not significant), and significant differences between categories are indicated by different letters for the post hoc tests.

	Two-way ANOVA	Post hoc analysis (Tukey's HSD test): Differences between seasons for each river				Post hoc analysis (Tukey's HSD test): Differences between rivers for each season				
			Spring	Summer /fall	Winter		Ob	Yenisey	Lena	Kolyma
DOC $\delta^{13}C$	River n.s.	Ob	a	a	a	Spring	a	a	a	a
	Season n.s.	Yenisey	b	ab	a	Summer/fall	a	a	a	a
	River x season $p = 0.021$	Lena	a	a	a	Winter	a	a	a	a
		Kolyma	a	a	a					
POC $\delta^{13}C$	River $p < 0.001$	Ob	a	ab	b	Spring	c	b	a	ab
	Season $p < 0.001$	Yenisey	a	b	a	Summer/fall	b	b	a	a
	River x season $p < 0.001$	Lena	a	a	b	Winter	b	a	b	b
		Kolyma	a	a	b					
DOC $\Delta^{14}C$	River $p < 0.001$	Ob	a	a	b	Spring	b	a	a	a
	Season $p < 0.001$	Yenisey	a	b	c	Summer/fall	b	b	a	b
	River x season $p = 0.016$	Lena	a	ab	b	Winter	b	ab	a	ab
		Kolyma	a	b	b					
POC $\Delta^{14}C$	River $p < 0.001$	Ob	a	a	a	Spring	ab	a	b	c
	Season $p = 0.052$	Yenisey	a	a	b	Summer/fall	a	ab	b	c
	River x season $p < 0.001$	Lena	ab	b	a	Winter	a	a	a	a
		Kolyma	ab	b	a					

Table S8. Fraction of organic carbon from permafrost and peat deposits in DOC and POC of Ob, Yenisey, Lena, and Kolyma in different seasons.

Table S8. Fraction of organic carbon from permafrost and peat deposits in DOC and POC of Ob, Yenisey, Lena, and Kolyma in different seasons. Values are means ± standard deviations, estimated using source apportionment with Markov chain Monte Carlo simulations based on $\Delta^{14}C$ values of DOC and POC as well as potential organic carbon sources. Best Estimate, Minimum, and Maximum represent model scenarios for permafrost and peat carbon.

		Best Estimate (Fraction of total)	Minimum (Fraction of total)	Maximum (Fraction of total)
DOC				
Ob	Spring	0.160 ± 0.052	0.114 ± 0.039	0.263 ± 0.073
Ob	Summer/fall	0.189 ± 0.060	0.135 ± 0.045	0.303 ± 0.085
Ob	Winter	0.312 ± 0.072	0.228 ± 0.054	0.479 ± 0.109
Yenisey	Spring	0.072 ± 0.043	0.050 ± 0.031	0.127 ± 0.066
Yenisey	Summer/fall	0.168 ± 0.059	0.119 ± 0.045	0.273 ± 0.086
Yenisey	Winter	0.253 ± 0.078	0.183 ± 0.058	0.398 ± 0.119
Lena	Spring	0.056 ± 0.035	0.032 ± 0.021	0.133 ± 0.070
Lena	Summer/fall	0.084 ± 0.043	0.049 ± 0.027	0.195 ± 0.081
Lena	Winter	0.116 ± 0.054	0.069 ± 0.034	0.254 ± 0.104
Kolyma	Spring	0.079 ± 0.039	0.046 ± 0.024	0.187 ± 0.074
Kolyma	Summer/fall	0.124 ± 0.045	0.073 ± 0.029	0.272 ± 0.082
Kolyma	Winter	0.163 ± 0.065	0.098 ± 0.040	0.345 ± 0.125
POC				
Ob	Spring	0.605 ± 0.063	0.452 ± 0.042	0.864 ± 0.075
Ob	Summer/fall	0.649 ± 0.071	0.484 ± 0.047	0.893 ± 0.070
Ob	Winter	0.650 ± 0.104	0.478 ± 0.068	0.862 ± 0.091
Yenisey	Spring	0.609 ± 0.062	0.448 ± 0.041	0.874 ± 0.072
Yenisey	Summer/fall	0.721 ± 0.080	0.535 ± 0.051	0.926 ± 0.055
Yenisey	Winter	0.882 ± 0.075	0.711 ± 0.079	0.957 ± 0.037
Lena	Spring	0.554 ± 0.050	0.345 ± 0.023	0.937 ± 0.048
Lena	Summer/fall	0.624 ± 0.058	0.394 ± 0.024	0.956 ± 0.037
Lena	Winter	0.510 ± 0.079	0.308 ± 0.038	0.873 ± 0.086
Kolyma	Spring	0.778 ± 0.060	0.493 ± 0.019	0.983 ± 0.016
Kolyma	Summer/fall	0.853 ± 0.060	0.539 ± 0.019	0.988 ± 0.012
Kolyma	Winter	0.713 ± 0.096	0.366 ± 0.037	0.952 ± 0.042

Table S9. Organic carbon export by Ob, Yenisey, Lena, and Kolyma in different seasons.

Table S9. Organic carbon export by Ob, Yenisey, Lena, and Kolyma in different seasons. Total organic carbon fluxes are described in previous publications (2, 3), and organic carbon fluxes from permafrost and peat deposits were calculated using source apportionment with Markov chain Monte Carlo simulations. Values are means ± standard deviations. Best Estimate, Minimum, and Maximum represent model scenarios for permafrost and peat carbon.

		Total organic carbon	Organic carbon from permafrost and peat deposits		
			Best Estimate	Minimum	Maximum
		(Tg year^{-1})	(Tg year^{-1})	(Tg year^{-1})	(Tg year^{-1})
DOC					
Ob	Spring	1.338 ± 0.149	0.216 ± 0.074	0.152 ± 0.056	0.351 ± 0.107
Ob	Summer/fall	2.171 ± 0.519	0.409 ± 0.166	0.294 ± 0.124	0.656 ± 0.245
Ob	Winter	0.609 ± 0.089	0.189 ± 0.052	0.139 ± 0.039	0.292 ± 0.079
Yenisey	Spring	2.924 ± 0.383	0.210 ± 0.133	0.145 ± 0.094	0.370 ± 0.204
Yenisey	Summer/fall	1.183 ± 0.199	0.198 ± 0.079	0.141 ± 0.059	0.322 ± 0.116
Yenisey	Winter	0.537 ± 0.092	0.136 ± 0.048	0.098 ± 0.036	0.214 ± 0.074
Lena	Spring	2.823 ± 0.613	0.057 ± 0.108	0.092 ± 0.066	0.371 ± 0.219
Lena	Summer/fall	2.350 ± 0.547	0.196 ± 0.114	0.115 ± 0.069	0.458 ± 0.224
Lena	Winter	0.508 ± 0.174	0.059 ± 0.036	0.035 ± 0.022	0.129 ± 0.070
Kolyma	Spring	0.449 ± 0.174	0.036 ± 0.023	0.021 ± 0.014	0.084 ± 0.047
Kolyma	Summer/fall	0.329 ± 0.095	0.041 ± 0.020	0.024 ± 0.012	0.089 ± 0.038
Kolyma	Winter	0.040 ± 0.022	0.007 ± 0.004	0.004 ± 0.003	0.014 ± 0.009
POC					
Ob	Spring	0.250 ± 0.030	0.151 ± 0.024	0.113 ± 0.017	0.216 ± 0.032
Ob	Summer/fall	0.260 ± 0.045	0.168 ± 0.035	0.126 ± 0.025	0.233 ± 0.044
Ob	Winter	0.063 ± 0.006	0.041 ± 0.008	0.030 ± 0.005	0.054 ± 0.008
Yenisey	Spring	0.141 ± 0.012	0.086 ± 0.011	0.064 ± 0.008	0.124 ± 0.014
Yenisey	Summer/fall	0.073 ± 0.009	0.053 ± 0.009	0.039 ± 0.006	0.068 ± 0.009
Yenisey	Winter	0.033 ± 0.003	0.029 ± 0.004	0.023 ± 0.003	0.032 ± 0.003
Lena	Spring	0.382 ± 0.096	0.212 ± 0.057	0.132 ± 0.034	0.357 ± 0.093
Lena	Summer/fall	0.422 ± 0.078	0.263 ± 0.055	0.167 ± 0.032	0.403 ± 0.077
Lena	Winter	0.011 ± 0.003	0.006 ± 0.002	0.003 ± 0.001	0.010 ± 0.003
Kolyma	Spring	0.072 ± 0.045	0.060 ± 0.032	0.038 ± 0.020	0.076 ± 0.040
Kolyma	Summer/fall	0.049 ± 0.015	0.042 ± 0.013	0.026 ± 0.008	0.048 ± 0.015
Kolyma	Winter	0.002 ± 0.000	0.001 ± 0.000	0.001 ± 0.000	0.002 ± 0.000

References

1. McClelland JW, et al. (2008) Development of a pan-arctic database for river chemistry. *Eos (Washington DC)* 89(24):217–218.
2. McClelland JW, et al. (2016) Particulate organic carbon and nitrogen export from major Arctic rivers. *Global Biogeochem Cycles* 30:629–643.
3. Holmes RM, et al. (2012) Seasonal and annual fluxes of nutrients and organic matter from large rivers to the Arctic Ocean and surrounding seas. *Estuaries and Coasts* 35(2):369–382.
4. Raymond PA, et al. (2007) Flux and age of dissolved organic carbon exported to the Arctic Ocean: A carbon isotopic study of the five largest arctic rivers. *Global Biogeochem Cycles* 21(4):GB4011.
5. Trumbore S (2009) Radiocarbon and soil carbon dynamics. *Annu Rev Earth Planet Sci* 37:47–66.
6. Andersson RA, Meyers P, Hornibrook E, Kuhry P, Mörth C-M (2012) Elemental and isotopic carbon and nitrogen records of organic matter accumulation in a Holocene permafrost peat sequence in the East European Russian Arctic. *J Quat Sci* 27(6):545–552.
7. Larsson A, Segerström U, Laudon H, Nilsson MB (2017) Holocene carbon and nitrogen accumulation rates in a boreal oligotrophic fen. *The Holocene* 27(6):811–821.
8. Hilasvuori E, et al. (2013) Temperature sensitivity of decomposition in a peat profile. *Soil Biol Biochem* 67:47–54.
9. Rask HM, Schoenau JJ (1993) ^{13}C natural abundance variations in carbonates and organic carbon from boreal forest wetlands. *Biogeochemistry* 22(1):23–35.
10. Normand AE, Smith AN, Clark MW, Long JR, Reddy KR (2017) Chemical composition of soil organic matter in a subarctic peatland: Influence of shifting vegetation communities. *Soil Sci Soc Am J* 81:41–49.
11. Krohn J, Lozanovska I, Kuzyakov Y, Parvin S, Dorodnikov M (2017) CH_4 and CO_2 production below two contrasting peatland micro-relief forms: An inhibitor and $\delta^{13}C$ study. *Sci Total Environ* 586:142–151.
12. Esmeijer-Liu AJ, Kürschner WM, Lotter AF, Verhoeven JTA, Goslar T (2012) Stable carbon and nitrogen isotopes in a peat profile are influenced by early stage diagenesis and changes in atmospheric CO_2 and N deposition. *Water, Air, Soil Pollut* 223(5):2007–2022.
13. Alewell C, Giesler R, Klaminder J, Leifeld J, Rollog M (2011) Stable carbon isotopes as indicators for environmental change in palsa peats. *Biogeosciences* 8(7):1769–1778.
14. Strauss J, et al. (2015) Organic-matter quality of deep permafrost carbon - a study from Arctic Siberia. *Biogeosciences* 12(7):2227–2245.
15. Fritz M, et al. (2015) Holocene ice-wedge polygon development in northern Yukon permafrost peatlands (Canada). *Quat Sci Rev* 147:279–297.
16. Lenz J, et al. (2016) Evidence of multiple thermokarst lake generations from an 11 800-year-old permafrost core on the northern Seward Peninsula, Alaska. *Boreas* 45(4):584–603.
17. Lenz J, et al. (2013) Periglacial landscape dynamics in the western Canadian Arctic: Results from a thermokarst lake record on a push moraine (Herschel Island, Yukon Territory). *Palaeogeogr Palaeoclimatol Palaeoecol* 381–382:15–25.
18. Wooller MJ, et al. (2012) Reconstruction of past methane availability in an Arctic Alaska wetland indicates climate influenced methane release during the past ~12,000 years. *J Paleolimnol* 48(1):27–42.
19. Schirrmeister L, et al. (2011) Sedimentary characteristics and origin of the Late Pleistocene Ice Complex on north-east Siberian Arctic coastal lowlands and islands - A review. *Quat Int* 241(1–2):3–25.
20. Vonk JE, et al. (2012) Activation of old carbon by erosion of coastal and subsea permafrost in Arctic Siberia. *Nature* 489(7414):137–140.

21. Tesi T, et al. (2016) Massive remobilization of permafrost carbon during post-glacial warming. *Nat Commun* 7:13653.
22. Andersson A, et al. (2015) Regionally-varying combustion sources of the January 2013 severe haze events over eastern China. *Environ Sci Technol* 49(4):2038–2043.
23. R Core Team (2018) R: A language and environment for statistical computing. Available at: https://www.r-project.org/.
24. Harrell FEJ (2018) Hmisc: Harrell Miscellaneous. *R Package version 41-1*. Available at: https://cran.r-project.org/package=Hmisc.
25. Pasek J (2018) weights: Weighting and weighted statistics. *R Package version 10*. Available at: https://cran.r-project.org/package=weights.
26. Li J, Ziegler S, Lane CS, Billings SA (2012) Warming-enhanced preferential microbial mineralization of humified boreal forest soil organic matter: Interpretation of soil profiles along a climate transect using laboratory incubations. *J Geophys Res Biogeosciences* 117(2):1–13.
27. Xu C, Guo L, Ping CL, White DM (2009) Chemical and isotopic characterization of size-fractionated organic matter from cryoturbated tundra soils, northern Alaska. *J Geophys Res Biogeosciences* 114(3):1–11.
28. Gentsch N, et al. (2015) Storage and transformation of organic matter fractions in cryoturbated permafrost soils across the Siberian Arctic. *Biogeosciences* 12(14):4525–4542.
29. Krab EJ, Berg MP, Aerts R, van Logtestijn RSP, Cornelissen JHC (2013) Vascular plant litter input in subarctic peat bogs changes Collembola diets and decomposition patterns. *Soil Biol Biochem* 63:106–115.
30. Schnecker J, et al. (2015) Microbial community composition shapes enzyme patterns in topsoil and subsoil horizons along a latitudinal transect in Western Siberia. *Soil Biol Biochem* 83:106–115.
31. Clemmensen KE, et al. (2013) Roots and associated fungi drive long-term carbon sequestration in boreal forest. *Science* 339(March):1615–1618.
32. Hugelius G, Routh J, Kuhry P, Crill P (2012) Mapping the degree of decomposition and thaw remobilization potential of soil organic matter in discontinuous permafrost terrain. *J Geophys Res Biogeosciences* 117(2):G02030.
33. Kaiser C, et al. (2007) Conservation of soil organic matter through cryoturbation in arctic soils in Siberia. *J Geophys Res Biogeosciences* 112(2):1–8.
34. Nowinski NS, Trumbore SE, Schuur EAG, MacK MC, Shaver GR (2008) Nutrient addition prompts rapid destabilization of organic matter in an arctic tundra ecosystem. *Ecosystems* 11(1):16–25.
35. Hartley IP, et al. (2012) A potential loss of carbon associated with greater plant growth in the European Arctic. *Nat Clim Chang* 2(12):875–879.
36. Schuur EAG, Trumbore SE, Mack MC, Harden JW (2003) Isotopic composition of carbon dioxide from a boreal forest fire: Inferring carbon loss from measurements and modeling. *Global Biogeochem Cycles* 17(1):1001.
37. Goncharov AA, Tsurikov SM, Potapov AM, Tiunov AV (2016) Short-term incorporation of freshly fixed plant carbon into the soil animal food web: field study in a spruce forest. *Ecol Res* 31(6):923–933.
38. Kielland K, Bryant JP (1998) Moose herbivory in taiga: effects on biogeochemistry and vegetation dynamics in primary succession. *Oikos* 82(2):377–383.
39. Kudrin AA, Tsurikov SM, Tiunov A V. (2015) Trophic position of microbivorous and predatory soil nematodes in a boreal forest as indicated by stable isotope analysis. *Soil Biol Biochem* 86:193–200.
40. Norris CE, Quideau SA, Oh SW (2016) Microbial utilization of double-labeled aspen litter in boreal aspen and spruce soils. *Soil Biol Biochem* 100:9–20.

41. Preston CM, Bhatti JS, Flanagan LB, Norris C (2006) Stocks, chemistry, and sensitivity to climate change of dead organic matter along the Canadian boreal forest transect case study. *Clim Change* 74(1–3):233–251.
42. Czimczik CI, Trumbore SE (2007) Short-term controls on the age of microbial carbon sources in boreal forest soils. *J Geophys Res Biogeosciences* 112(3):1–8.
43. Dutta K, Schuur EAG, Neff JC, Zimov SA (2006) Potential carbon release from permafrost soils of Northeastern Siberia. *Glob Chang Biol* 12(12):2336–2351.
44. Gundelwein A, et al. (2007) Carbon in tundra soils in the Lake Labaz region of arctic Siberia. *Eur J Soil Sci* 58(5):1164–1174.
45. Rodionow A, Flessa H, Kazansky O, Guggenberger G (2006) Organic matter composition and potential trace gas production of permafrost soils in the forest tundra in northern Siberia. *Geoderma* 135:49–62.
46. Höfle S, Rethemeyer J, Mueller CW, John S (2013) Organic matter composition and stabilization in a polygonal tundra soil of the Lena Delta. *Biogeosciences* 10(5):3145–3158.
47. Jasinski JPP, et al. (1998) Holocene environmental history of a peatland in the Lena River valley, Siberia. *Can J Earth Sci* 35(6):637–648.
48. Palmtag J, et al. (2015) Storage, landscape distribution, and burial history of soil organic matter in contrasting areas of continuous permafrost. *Arctic, Antarct Alp Res* 47(1):71–88.
49. Palmtag J, et al. (2016) Controls on the storage of organic carbon in permafrost soil in northern Siberia. *Eur J Soil Sci* 67(4):478–491.
50. Siewert MB, et al. (2015) Comparing carbon storage of Siberian tundra and taiga permafrost ecosystems at very high spatial resolution. *J Geophys Res Biogeosciences* 120:1973–1994.
51. Weiss N, et al. (2016) Thermokarst dynamics and soil organic matter characteristics controlling initial carbon release from permafrost soils in the Siberian Yedoma region. *Sediment Geol* 340:38–48.
52. Bird MI, et al. (2002) Soil carbon inventories and carbon-13 on a latitude transect in Siberia. *Tellus, Ser B Chem Phys Meteorol* 54(5):631–641.
53. Schulze ED, Lapshina E, Filippov I, Kuhlmann I, Mollicone D (2015) Carbon dynamics in boreal peatlands of the Yenisey region, western Siberia. *Biogeosciences* 12(23):7057–7070.
54. Andreev AA, Klimanov VA, Sulerzhitsky LD (2001) Vegetation and climate history of the Yana River lowland, Russia, during the last 6400 yr. *Quat Sci Rev* 20(1–3):259–266.
55. MacDonald GM, et al. (2006) Rapid early development of circumarctic peatlands and atmospheric CH_4 and CO_2 variations. *Science* 314(October):285–288.
56. Andreev AA, et al. (2002) Late Pleistocene and Holocene vegetation and climate on the Taymyr Lowland, Northern Siberia. *Quat Res* 57(01):138–150.
57. Peteet D, Andreev A, Bardeen W, Mistretta F (1998) Long-term Arctic peatland dynamics, vegetation and climate history of the Pur-Taz region, western Siberia. *Boreas* 27(2):115–126.
58. Vasil'chuk YK, Jungner H, Vasil'chuk AC (2001) ^{14}C dating of peat and $\delta^{18}O$-δD in ground ice from Northwest Siberia. *Radiocarbon* 43(2):527–540.
59. Walter Anthony KM, et al. (2014) A shift of thermokarst lakes from carbon sources to sinks during the Holocene epoch. *Nature* 511(7510):452–456.
60. Andreev AA, et al. (2009) Weichselian and Holocene palaeoenvironmental history of the Bol'shoy Lyakhovsky Island, New Siberian Archipelago, Arctic Siberia. *Boreas* 38(1):72–110.
61. Opel T, et al. (2017) Ground-ice stable isotopes and cryostratigraphy reflect late Quaternary palaeoclimate in the Northeast Siberian Arctic (Oyogos Yar coast, Dmitry Laptev Strait). *Clim Past* (13):587–611.

62. Schirrmeister L, et al. (2008) Periglacial landscape evolution and environmental changes of Arctic lowland areas for the last 60000 years (western Laptev Sea coast, Cape Mamontov Klyk). *Polar Res* 27(2):249–272.
63. Schleusner P, Biskaborn BK, Kienast F, Wolter J, Subetto D (2015) Basin evolution and palaeoenvironmental variability of the thermokarst lake El'gene-Kyuele , Arctic Siberia. *Boreas* 44:216–229.
64. Shilo NA, Lozhkin AV, Anderson PM (2007) Radiocarbon dates of evolution cycles of thermokarst lakes on the Kolyma Lowland. *Dokl Earth Sci* 413(2):259–261.
65. Wetterich S, et al. (2009) Eemian and Late Glacial/Holocene palaeoenvironmental records from permafrost sequences at the Dmitry Laptev Strait (NE Siberia, Russia). *Palaeogeogr Palaeoclimatol Palaeoecol* 279(1–2):73–95.
66. Andreev AA, et al. (2002) Paleoenvironmental changes in Northeastern Siberia during the Late Quaternary - Evidence from pollen records of the Bykovsky Peninsula. *Polarforschung* 70(1–2):13–25.
67. Ashastina K, Schirrmeister L, Fuchs M, Kienast F (2017) Palaeoclimate characteristics in interior Siberia of MIS 6-2: First insights from the Batagay permafrost mega-thaw slump in the Yana Highlands. *Clim Past* 13(7):795–818.
68. Schwamborn G, Rachold V, Grigoriev MN (2002) Late Quaternary sedimentation history of the Lena Delta. *Quat Int* 89:119–134.
69. Grosse G, et al. (2007) Geological and geomorphological evolution of a sedimentary periglacial landscape in Northeast Siberia during the Late Quaternary. *Geomorphology* 86:25–51.
70. Murton JB, et al. (2015) Palaeoenvironmental interpretation of Yedoma silt (Ice Complex) deposition as cold-climate loess, Duvanny Yar, Northeast Siberia. *Permafr Periglac Process* 26(3):208–288.
71. Müller S, Bobrov AA, Schirrmeister L, Andreev AA, Tarasov PE (2009) Testate amoebae record from the Laptev Sea coast and its implication for the reconstruction of Late Pleistocene and Holocene environments in the Arctic Siberia. *Palaeogeogr Palaeoclimatol Palaeoecol* 271(3–4):301–315.
72. Péwé TL, Journaux A, Stuckenrath R (1977) Radiocarbon dates and late-Quaternary stratigraphy from Mamontova Gora, unglaciated central Yakutia, Siberia, U.S.S.R. *Quat Res* 8:51–63.
73. Schirrmeister L, Siegert C, Kunitzky VV, Grootes PM, Erlenkeuser H (2002) Late Quaternary ice-rich permafrost sequences as a paleoenvironmental archive for the Laptev Sea Region in Northern Siberia. *Int J Earth Sci* 91(1):154–167.
74. Schirrmeister L, et al. (2003) Late Quaternary history of the accumulation plain north of the Chekanovsky Ridge (Lena Delta, Russia): a multidisciplinary approach. *Polar Geogr* 27(4):277–319.
75. Vasil'chuk YK, Vasil'chuk AC (1997) Radiocarbon dating and oxygen isotope variations in late Pleistocene. *Permafr Periglac Process* 8:335–345.
76. Vasil'chuk YK, Vasil'chuk AC (1998) Oxygen-isotope and ^{14}C data associated with late Pleistocene syngenetic ice-wedges in mountains of Magadan Region, Siberia. *Permafr Periglac Process* 9(2):177–183.
77. Vasil'chuk YK, van der Plicht J, Jungner H, Sonninen E, Vasil'chuk AC (2000) First direct dating of Late Pleistocene ice-wedges by AMS. *Earth Planet Sci Lett* 179:237–242.
78. Vasil'chuk YK (2005) Heterochroneity and heterogeneity of the Duvanny Yar Edoma. *Dokl Earth Sci* 402(4):568–573.
79. Wetterich S, et al. (2008) Palaeoenvironmental dynamics inferred from late Quaternary permafrost deposits on Kurungnakh Island, Lena Delta, Northeast Siberia, Russia. *Quat Sci Rev* 27(15–16):1523–1540.
80. Wetterich S, et al. (2016) Ice Complex permafrost of MIS5 age in the Dmitry Laptev

Strait coastal region (East Siberian Arctic). *Quat Sci Rev* 147:298–311.
81. Zech M, et al. (2008) Characterisation and palaeoclimate of a loess-like permafrost palaeosol sequence in NE Siberia. *Geoderma* 143(3–4):281–295.
82. HH: Statistical analysis and data display: Heiberger and Holland (2018) *R Package version 31-35*. Available at: https://cran.r-project.org/package=HH.
